# Molecular and spatial signatures of mouse brain aging at single-cell resolution

**DOI:** 10.1016/j.cell.2022.12.010

**Published:** 2022-12-28

**Authors:** William E. Allen, Timothy R. Blosser, Zuri A. Sullivan, Catherine Dulac, Xiaowei Zhuang

**Affiliations:** 1 Society of Fellows, Harvard University, Cambridge, MA 02138, USA; 2 Howard Hughes Medical Institute, Department of Chemistry and Chemical Biology, and Department of Physics, Harvard University, Cambridge, MA 02138, USA; 3 Howard Hughes Medical Institute, Department of Molecular and Cellular Biology, Harvard University, Cambridge, MA 02138, USA; 4 Center for Brain Science, Harvard University, Cambridge, MA 02138, USA; 5 Lead contact

## Abstract

The diversity and complex organization of cells in the brain have hindered systematic characterization of age-related changes in its cellular and molecular architecture, limiting our ability to understand the mechanisms underlying its functional decline during aging. Here, we generated a high-resolution cell atlas of brain aging within the frontal cortex and striatum using spatially resolved single-cell transcriptomics and quantified changes in gene expression and spatial organization of major cell types in these regions over the mouse lifespan. We observed substantially more pronounced changes in cell state, gene expression, and spatial organization of non-neuronal cells over neurons. Our data revealed molecular and spatial signatures of glial and immune cell activation during aging, particularly enriched in the subcortical white matter, and identified both similarities and notable differences in cell-activation patterns induced by aging and systemic inflammatory challenge. These results provide critical insights into age-related decline and inflammation in the brain.

## In brief

Single-cell transcriptome imaging and sequencing of aging mouse brain reveals spatially dependent and cell-type-specific changes in cellular state shared in part with systemic inflammatory challenge.

## Graphical Abstract

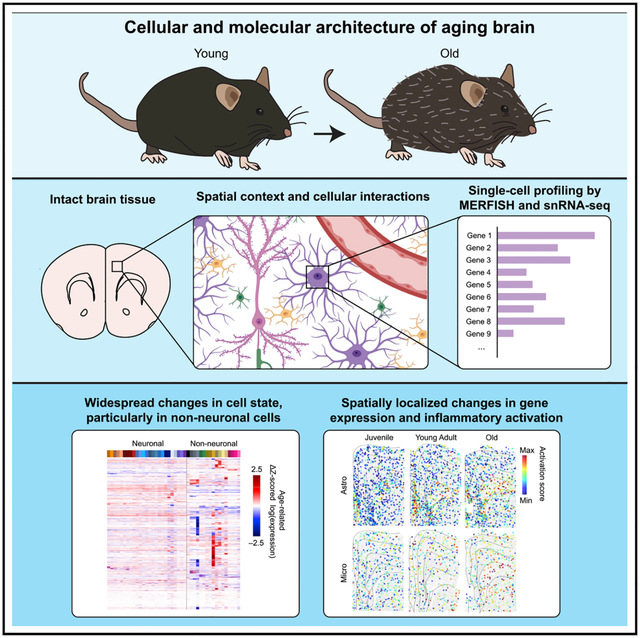

## INTRODUCTION

The mammalian brain exhibits remarkable stability over periods ranging from years to decades.^1^ Due to the brain’s limited regenerative abilities, neurons must faithfully perform their function for the entire lifetime of an animal. However, as the animals age, this longevity of neurons makes the brain sensitive to the accumulation of damage over time.^1^ This neuronal damage, in combination with age-dependent changes in non-neuronal cells that support neural circuit function, is thought to cause the decline of brain function and the increase in the prevalence of neurodegenerative disorders associated with aging.^1–3^

A prominent hypothesis underlying brain aging suggests that changes in neuronal and synaptic functions associated with age and neurodegeneration are the result of disruptions to the brain’s homeostatic environment.^4–6^ Neurons are supported by a host of non-neuronal cells, each maintaining different aspects of the tissue homeostasis.^7^ For example, oligodendrocytes myelinate axons and provide metabolic support to neurons; astrocytes provide trophic and ionic support to neurons and modulate synaptic function; and microglia provide immune surveillance, synaptic pruning, and debris removal by phagocytosis.^8–15^ Brain injury, infection, and neurodegeneration have been shown to trigger inflammatory activation of these resident non-neuronal cell types and recruit peripheral immune cells, resulting in both protective and deleterious effects for neighboring neurons.^6,11,13,15,16^

Recent transcriptomic studies of normal brain aging^17–20^ and neurodegenerative disease,^21–24^ as well as studies focusing on specific non-neuronal cell types such as astrocytes,^25–27^ microglia,^28,29^ and endothelial cells,^30^ have further highlighted a role for inflammatory activation in aging-related decline. In particular, reactive states that are typically triggered in microglia and astrocytes during infection or injury also emerge over the course of normal aging.

While these studies suggest broad age-related disruptions to brain homeostasis that manifest in a variety of cell types, they also raise many questions. For example, how do the molecular signatures and spatial organization of different cell types and cell states change over aging, and how do these changes relate to age-induced inflammatory activation? How are activated cells spatially distributed, and does this activation depend on environmental factors and cell-cell communications? How does age-induced inflammation relate to systemic inflammatory response? Answering these questions is challenging, as the brain’s enormous cellular and molecular complexity has so far prevented a comprehensive understanding of the changes in brain architecture over an animal’s lifetime.

Here, we performed a systematic characterization of the changes in molecular signatures and spatial organizations of cells during brain aging by using an experimental approach that combines a single-cell transcriptome imaging method, multiplexed error-robust fluorescent *in situ* hybridization (MERFISH),^31^ with single-nucleus RNA sequencing (snRNAseq).^32^ This approach allowed us to profile gene expression and identify cell types and states in the mouse frontal cortex and stratum, thus generating a spatially resolved cell atlas of these regions across different ages. This high-resolution cell atlas revealed age-related changes in both neurons and non-neuronal cells and uncovered molecular and spatial signatures of glial and immune cell activation during aging. Comparison with changes induced by lipopolysaccharide (LPS) further revealed previously unknown differences in non-neuronal cell activation induced by aging and by systemic inflammatory challenge.

## RESULTS

### Spatially resolved single-cell transcriptomic profiling of the aging brain

**Figure 1.**
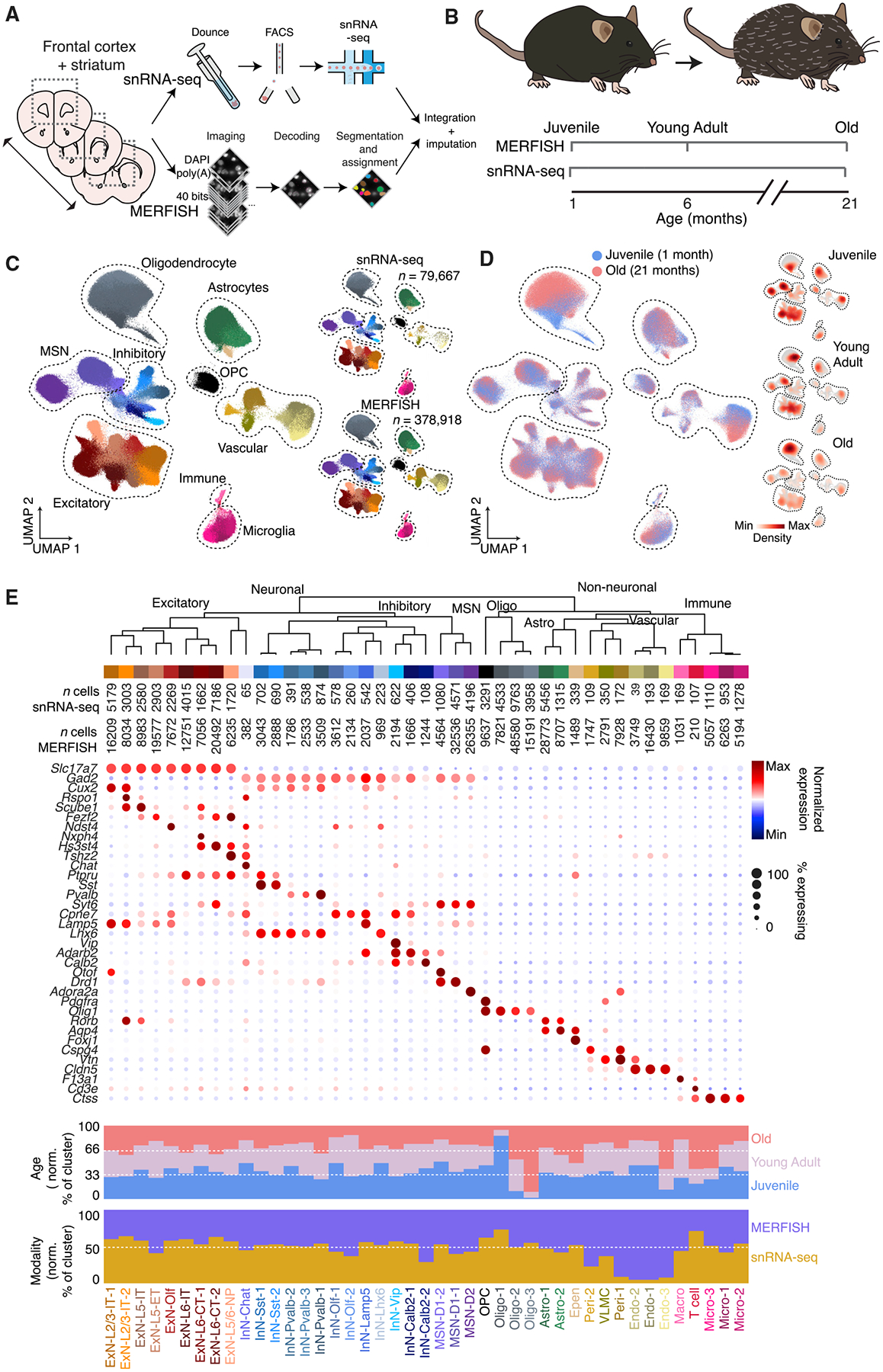
Spatially resolved single-cell transcriptomic profiling of the mouse frontal cortex and striatum across ages (A) Profiling of the mouse frontal cortex and striatum via integrated snRNA-seq and MERFISH analyses. (B) Sampling time points for snRNA-seq and MERFISH measurements across the lifespan of mice. (C) (Left) uniform manifold approximation (UMAP) visualization of cells from all timepoints, from both snRNA-seq and MERFISH measurements. (Right) separate UMAP of cells measured by snRNA-seq (top) and MERFISH (bottom). Cells are colored by cell-type assignment. (D) (Left) as in (C) but with cells colored by age. Only the juvenile and old time points are shown. (Right) Individual UMAP plots, shown as the density of cells at each time point overlaid on total cell population across all three ages (gray). (E) Molecularly defined cell types determined from integrated snRNA-seq and MERFISH clustering analysis. (Top) dendrogram of the hierarchical relationship among clusters and number of measured cells per cluster in snRNA-seq and MERFISH. (Middle) expression of example marker genes for different cell types. (Bottom) fraction of cells per cluster by age and by modality, normalized to sampling depth such that equal representation in each condition will have the same fraction. See also Figures S1 and S2 and Tables S1–S3.

We performed snRNA-seq measurements to probe the transcriptomic profiles of individual cells from the frontal cortex and striatum of mice at two different ages, ~1 month (4-week-old, juvenile) and ~21 months (90-week-old, old) postnatal (Figures 1A and 1B). These brain regions have been previously shown to be susceptible to age-related neurodegenerative diseases.^33,34^ We sequenced ~50,000 nuclei from two female animals at each age and performed unsupervised clustering analysis of the ~80,000 cells that passed quality control (Figures S1A–S1F).

We then selected two sets of genes for spatially resolved single-cell transcriptomic measurements by MERFISH based on the snRNA-seq results (Figure 1A): (1) cell-type markers that were differentially expressed between cell clusters determined by snRNA-seq and (2) aging-related genes that were differentially expressed between the two ages in individual cell clusters. In addition, we selected previously known cell-type marker genes that define major neuronal, glial, and immune cell types and genes previously reported to be highly upregulated in various cell types during aging. Together, these resulted in a total of 212 cell-type markers and 204 aging-related genes, which we imaged in the same tissue sections through two back-to-back MERFISH runs, each with a 20-bit barcoding scheme (STAR Methods and Tables S1–S3).

We imaged these 416 genes in the frontal cortex and striatum of mice across three different ages, ~1 month (4-week-old, juvenile), ~6 months (24-week-old, young adult), and ~21 months (90-week-old, old) postnatal, including 3–5 female animals at each age (Figure 1B). A total of ~400,000 cells were imaged and passed quality control. The average expression levels of individual genes measured by MERFISH showed good correlation with results from bulk RNA sequencing and were highly reproducible between biological replicates (Figures S1G–S1I). We then co-embedded the MERFISH and snRNA-seq data, including all measured cells, using the Harmony algorithm^35^ and performed an integrated clustering analysis across these two data modalities (Figures 1C and 1D), which showed good correspondence with the clustering results from the snRNA-seq data alone (Figure S1J). The integrated analysis resulted in a total of 43 neuronal and non-neuronal cell types (Figure 1E). Compared to our previous MERFISH results of the mouse cortex^36^ and striatum,^37^ cell clusters were dissected here at a lower granularity to capture age-related changes of major cell types. The neuronal clusters included layer-specific excitatory neuronal cell types (ExN) in the cortex (ExN-L2/3-IT, ExN-L5-IT, ExN-L5-ET, ExN-L5/6-NP, ExN-L6-IT, and ExN-L6-CT), inhibitory neuronal cell types (InN) in the cortex marked by canonical inhibitory neuronal markers (*Sst, Pvalb, Lamp5*, and *Vip*), excitatory and inhibitory neurons in the subcortical olfactory areas (ExN-Olf and InN-Olf*), Drd1+ *(D1) or *Drd2+ *(D2) medium spiny neurons (MSN), *Lhx6+* or *Chat+* interneurons in the striatum, as well as spatially dispersed *Calb2*+interneurons. The non-neuronal clusters include oligodendrocytes (Oligo), oligodendrocyte precursor cells (OPCs), astrocytes (Astro), ependymal cells (Epen), pericytes (Peri), vascular leptomeningeal cells (VLMCs), endothelial cells (Endo), microglia (Micro), macrophages (Macro), and T cells. snRNA-seq and MERFISH data co-embedded well with each other, and the vast majority of cell clusters were well represented in both datasets with high correspondence in gene expression between the clusters from each dataset (Figures 1C, 1E, and S1K). However, we note that some vascular cell types (pericytes and endothelial cells) were poorly sampled by snRNA-seq (Figure 1E).

A different integration method (Allcools)^38^ gave similar clustering results (Figure S1L). Performing integration using a balanced ratio of snRNA-seq and MERFISH cells (where the MERFISH cells were randomly subsampled to ~80,000 cells) also gave similar clustering results, with the exception of a few rare cell types that were not sampled well when the number of MERFISH cells was reduced (Figure S1M). We thus included all MERFISH cells in the integration for further analysis.

This integration also allowed us to impute genome-wide expression profiles for individual cells measured by MERFISH using the transcriptomic profiles of neighboring snRNA-seq cells in the gene-expression space (STAR Methods). As a validation, the imputed spatial distributions of the genes showed good agreement with both the results directly measured by MERFISH and the results from Allen brain *in situ* hybridization atlas (Figure S2), and both integration methods generated similar imputation results (Figure S2).

### Age-related changes in cell state and composition

**Figure 2.**
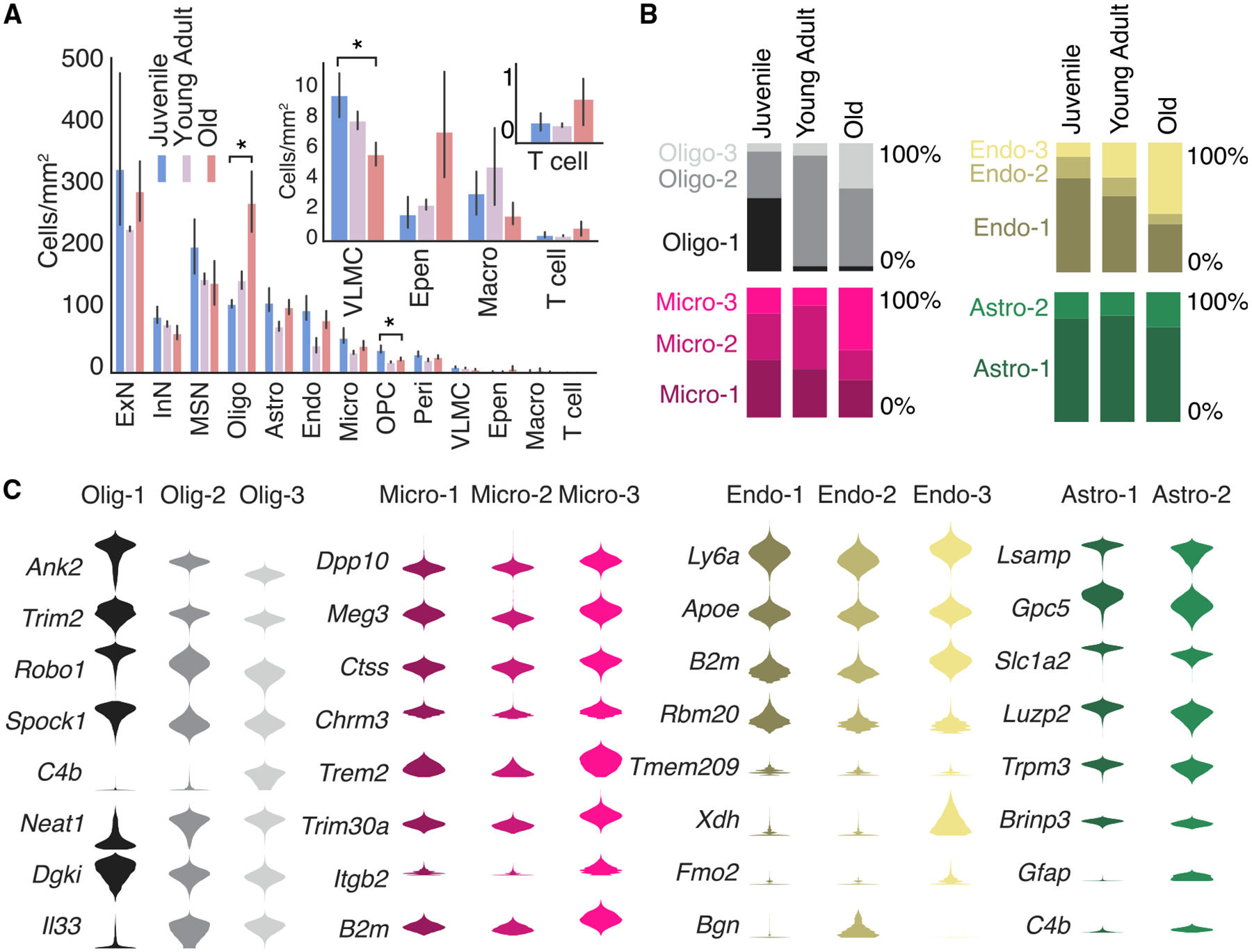
Changes in cell-type and cell-state composition of the mouse frontal cortex and striatum across ages (A) Density of different major cell types (in cells/mm^2^) across the three ages. Inset shows magnified view of lower abundance cell types. * indicates FDR-adjusted p-value < 0.05 (independent sample t-test) in the difference between juvenile and old animals. Data are presented as mean ±95% confidence interval. (B) Fraction of cells that belong to different states of oligodendrocytes, microglia, endothelial cells, and astrocytes across different ages. (C) Violin plot of expression of example genes across different states of oligodendrocytes, microglia, endothelial cells, and astrocytes. See also Tables S1–S3.

We next analyzed how the cell composition and cell state in these brain regions changed across the three ages based on the MERFISH data. The neuronal clusters did not exhibit any significant change in abundance (Figure 2A). By contrast, several non-neuronal cell types exhibited substantial age-dependent changes in the overall abundance and/or the relative proportions of cells in different states within a specific cell type (Figures 1E, 2A, and 2B). In particular, the abundance of oligodendrocytes increased and that of OPCs decreased substantially as the animal aged (Figure 2A). Among the three states of oligodendrocytes, the Oligo-1 cluster was predominant in juvenile animals and reduced to nearly non-existent in young adult and old animals, Oligo-2 was predominant in young adult animals and decreased in abundance in old animals, while Oligo-3 emerged in old animals (Figure 2B). The aging-related cluster Oligo-3 exhibited substantial upregulation of *C4b* (Figure 2C), a complement protein of the innate immunity system, and interleukin 33 (*Il33*) (Figure 2C), a cytokine involved in inflammatory and innate immune response,^39^ consistent with previous results.^17^ These results suggest an initial maturation and proliferation of oligodendrocytes, likely a result of late-stage development, followed by inflammatory activation of matured oligodendrocytes with aging. Microglia, endothelial cells, and astrocytes also showed an age-dependent shift in population among different cell states. For example, Micro-1 and Endo-1 were enriched in juvenile animals, Micro-3 and Endo-3 were enriched old animals, and Astro-2 showed increased in abundance in old animals (Figure 2B). These aging-related cell states exhibit upregulation of genes (e.g., *B2m* and *Trem2* in Micro-3, *Xdh* in Endo-3, and *Gfap* and *C4b* in Astro-2) (Figure 2C) that have been previously shown to be enriched in microglia, endothelial cells, and astrocytes activated by inflammation and/or aging.^17,25,26,29,30,40^ Consistent with T cell infiltration into the aging brain,^41^ we also observed a substantial increase in the abundance of T cells in old animals (Figure 2A), although the change did not reach statistical significance due to the small number of cells detected in this rare cell population.

### Age-related changes in the spatial distribution of individual cell types and cell states

**Figure 3.**
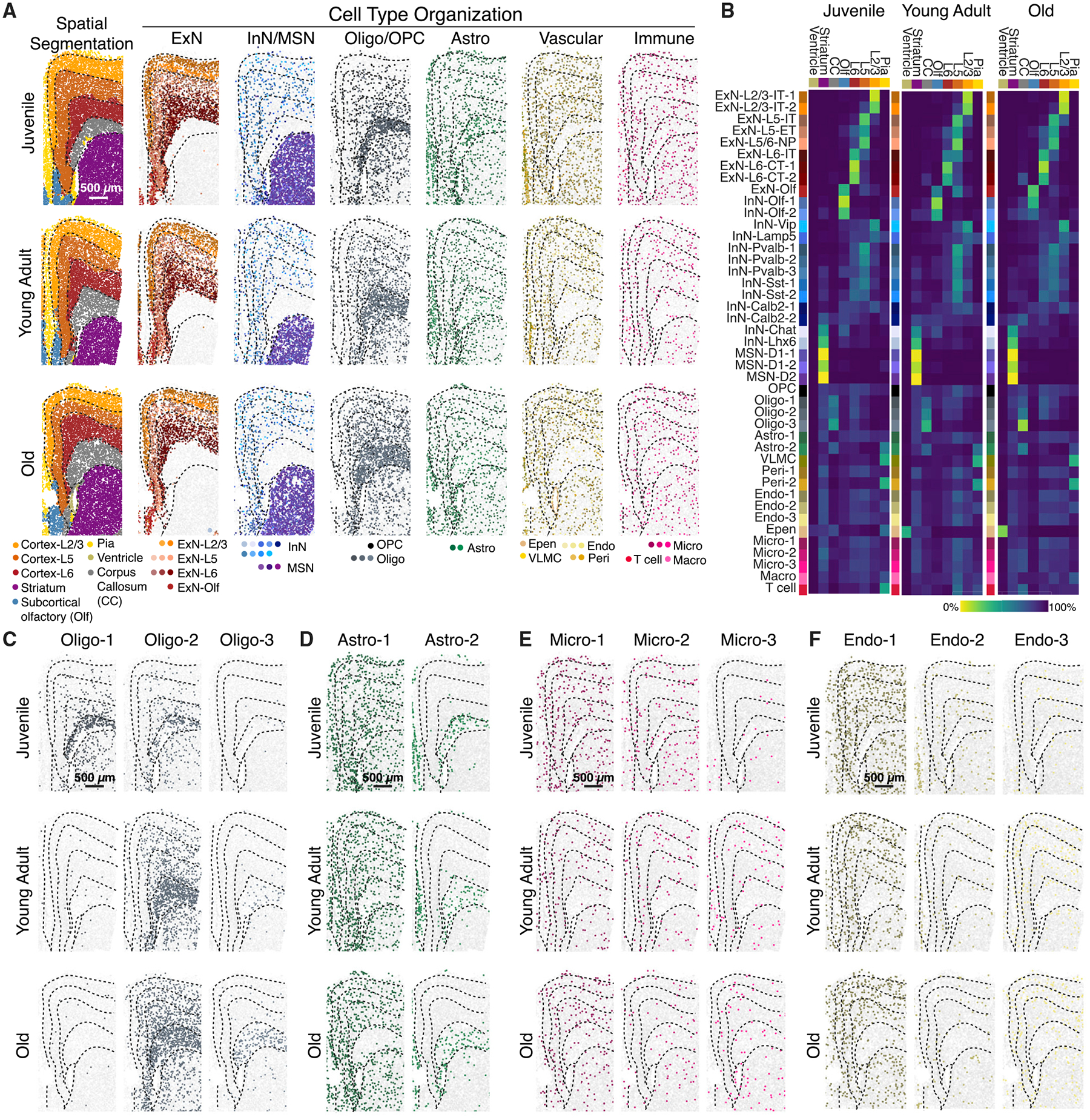
Spatial organization of cells in the mouse frontal cortex and striatum across ages (A) (Left) spatial segmentation of anatomical regions. (Right) spatial organization of major cell types at the three different ages, colored by cluster identity. Dashed lines outlining anatomical regions were manually traced from spatial segmentation. Scale bar: 500 μm. (B) Fraction of cells resided in individual anatomical regions for each cell cluster at the three different ages. CC: corpus callosum. Olf: subcortical olfactory areas. The lower abundance of ependymal cells in younger animals may be due to classification as molecularly similar astrocytes or loss of ventricle surface during tissue sectioning. (C–F) Spatial organizations of oligodendrocyte (C), astrocyte (D), microglial (E), and endothelial (F) clusters at different ages. Scale bar: 500 μm. See also Figure S3 and Tables S1–S3.

The MERFISH data further allowed us to map the spatial organization of individual cell types across different ages. To visualize the overall spatial organization of cells, we performed hierarchical clustering of cells based on the cell composition in their spatial neighborhood (STAR Methods) and the resulting spatial clusters naturally segmented the imaged area into known anatomical structures, including the pia, cortical layers, corpus callosum, striatum, ventricle, and subcortical olfactory regions (Figure 3A). As expected, different excitatory clusters adopted laminar distributions in the cortex and medium spiny neurons were localized to the striatum (Figures 3A and 3B); oligodendrocytes were enriched in the corpus callosum, VLMCs and a specific pericyte cluster (Peri-2) were enriched in the pia, and ependymal cells were localized around the ventricle, whereas OPCs, astrocytes, microglia, and endothelial cells were distributed largely uniformly throughout the imaged regions (Figures 3A and 3B).

Interestingly, although the overall spatial organization of neuronal cell types appeared similar across different ages (Figures 3A and 3B), some non-neuronal cells showed changes in anatomical enrichment with age. For example, the oligodendrocyte state that emerged in old animals (Oligo-3) appeared nearly exclusively in the corpus callosum, whereas Oligo-1 and Oligo-2, albeit being enriched in the corpus callosum, could be also found throughout the imaged regions (Figure 3C). Likewise, the aging-related Astro-2 cluster primarily appeared in the corpus callosum, whereas Astro-1 adopted a complementary distribution depleted in corpus callosum in young-adult and old animals (Figure 3D), suggesting a shift in astrocytic cell state in the corpus callosum as the animal aged. Different microglial and endothelial clusters, on the other hand, were more or less evenly distributed throughout all anatomical regions (Figures 3E and 3F).

In addition, certain non-neuronal cell types exhibited a tendency to be spatially colocalized. Specifically, vascular cells (endothelial cells, pericytes, and VLMCs) showed a tendency to be proximal to each other, and this tendency increased with age, primarily between juvenile and young-adult animals (Figure S3), which is likely a reflection of the maturation of vascular structures during late-stage development or activity-dependent growth and/or remodeling of vascular structures.^8^ Moreover, macrophages tended to be enriched near vascular cells and this tendency also increased with age, but primarily between young-adult and old animals (Figure S3), likely due to an aging-related inflammatory response. A similar trend was also observed for microglia, albeit to a lesser degree (Figure S3).

### Age-related changes in the gene-expression profiles of individual cell types

**Figure 4.**
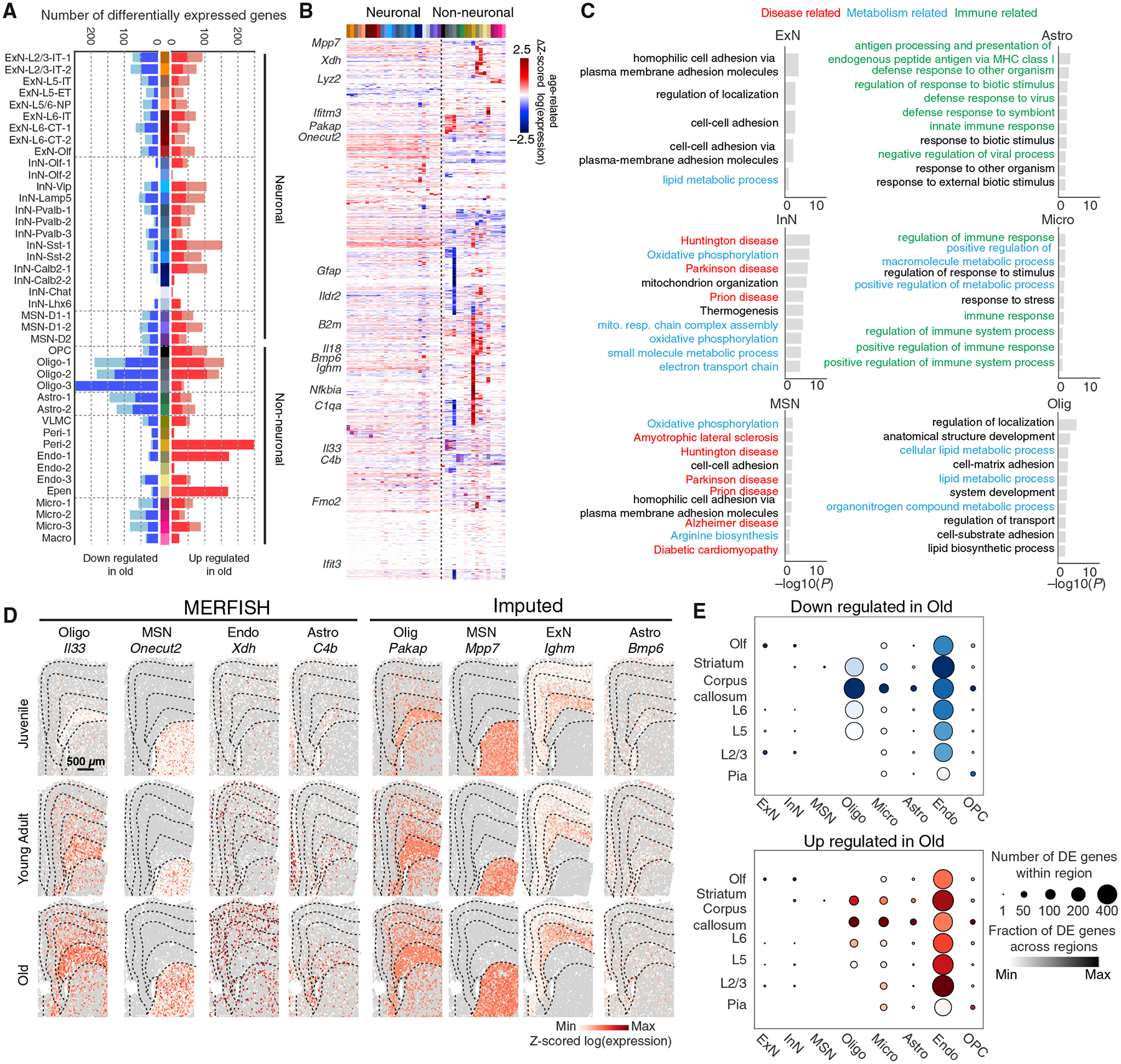
Cell-type-specific changes in molecular signatures across ages (A) Number of differentially expressed (DE) genes between juvenile and old animals in individual cell clusters, with genes up- and downregulated with age shown in red and blue bars, respectively. DE genes were defined as genes with age-related change in log(gene expression) > 2 (light colored bars) or >2.5 (dark colored bars) and FDR-adjusted p-value < 0.05 between the two ages. (B) Age-related change in Z-scored log(gene expression) between juvenile and old animals for DE genes in different cell clusters. (C) −log_10_(p-value) of enrichment for gene ontology (GO) biological process terms and Kyoto Encyclopedia of Genes and Genomes (KEGG) terms enriched among DE genes with an age-related change in log(gene expression) > 2 and FDR-adjusted p-value < 0.05 between the two ages (juvenile and old). Only top 10 (or fewer) GO or KEGG terms with p-value < 0.05 are listed for each major cell class. (D) Spatial maps of examples of DE genes across the three different ages, showing the expression of each gene within the indicated cell type. Gray spots indicate all other cells of other types.. Scale bar: 500 μm. (E) Quantification of the number of DE genes for each major cell type as a function of spatial location using imputed gene expression data derived from Harmony integration. DE genes with an age-related change in log(gene expression) > 2 and FDR-adjusted p-values < 0.05 are considered. Size of dot indicates total number of DE genes for a particular cell type within a region, and color shade of dot indicates the fraction within a particular region of the total number of DE genes that are differentially expressed across all regions, plotted on a relative scale of minimum fraction to maximum fraction across all regions. See also Figures S2 and S4 and Tables S1–S3.

Next, we examined how genome-wide expression profiles of individual cell types changed with age. To this end, we determined the number of genes that were differentially expressed between juvenile and old animals in individual neuronal and non-neuronal cell types based on the snRNA-seq data (Figure 4A). Non-neuronal cell types tended to exhibit a greater number of age-dependent differentially expressed genes than neurons (Figure 4A). Many age-dependent genes were differentially expressed in a cell-type-specific manner (Figure 4B). Gene ontology (GO) and KEGG (Kyoto Encyclopedia of Genes and Genomes) enrichment analysis showed that genes upregulated with age in neurons, in particular in inhibitory neurons, were enriched in pathways associated with neurodegenerative diseases, oxidative response, and mitochondria function, whereas genes upregulated with age in non-neuronal cells tended to be associated with inflammatory and immune response (Figure 4C), consistent with the previously observed broad upregulation of oxidative stress and immune pathways in the aging brain.^17–19,42^ Specifically, age-upregulated genes in non-neuronal cells included cytokines (e.g., *Il33* and *Il18* in oligodendrocytes), complement proteins (e.g., *C4b* in astrocytes and oligodendrocytes), and proteins involved in interferon response (e.g., *Ifit3* and *Ifitm3* in ependymal cells and pericytes) (Figure 4B).

Imputed genome-wide expression profiles of the cells measured by MERFISH allowed us the determine the spatial distributions of all genes across different ages. Many of the age-upregulated genes exhibited specific spatial patterns (see examples in Figure 4D). We systematically quantified the number of genes differentially expressed between juvenile and old animals for each major cell type in individual anatomical regions and observed spatial heterogeneity in the total number of genes upregulated or downregulated with age even among cells of the same type (Figures 4E and S2E). In particular, oligodendrocytes, astrocytes, and microglia all exhibited the greatest number of differentially expressed genes with age in the white matter of the corpus callosum relative to other anatomical regions.

To further investigate age-related changes in gene expression in non-neuronal cell types, we performed gene-gene correlation analysis and identified groups of genes within each cell type whose expression showed correlated variations with each other and hence likely belong to the same gene regulatory networks. This analysis revealed many groups of genes that showed correlated expression, referred to here as gene modules, within oligodendrocytes, astrocytes, and microglia (Figure S4). Many of these gene modules showed up- or downregulation in expression with age (Figure S4). GO or KEGG term analysis showed that many of these modules were related to either development or immune response, often capturing cell-type-specific functions. These results suggest the presence of specific gene regulatory networks that function in a cell-type-specific and age-dependent manner.

Of note, since the snRNA-seq measurements were performed on juvenile and old animals only, but not on young-adult animals, the observed changes in gene expression described in this section could thus be due to either late development or aging. Indeed, both types of changes were observed.

### Age-dependent activation of glial and immune cells

For microglia and astrocytes, we observed that the genes highly upregulated with age overlapped substantially with genes that have been previously reported as being upregulated in the activated (or “reactive”) state of these cell types (e.g., *Gfap* and *C4b* for astrocytes; *B2m* and *Lyz2* for microglia).^25,29,40,43^ These activated astrocytes and microglia have been observed in both healthy and diseased brains, often responding to brain injury, inflammation, or degeneration.^6,11,13,15,16,44^ Microglial and astrocytic activation has also been reported in aged rodent and human brains,^25–29^ but how such activation depends on the spatial context remains unclear.

**Figure 5.**
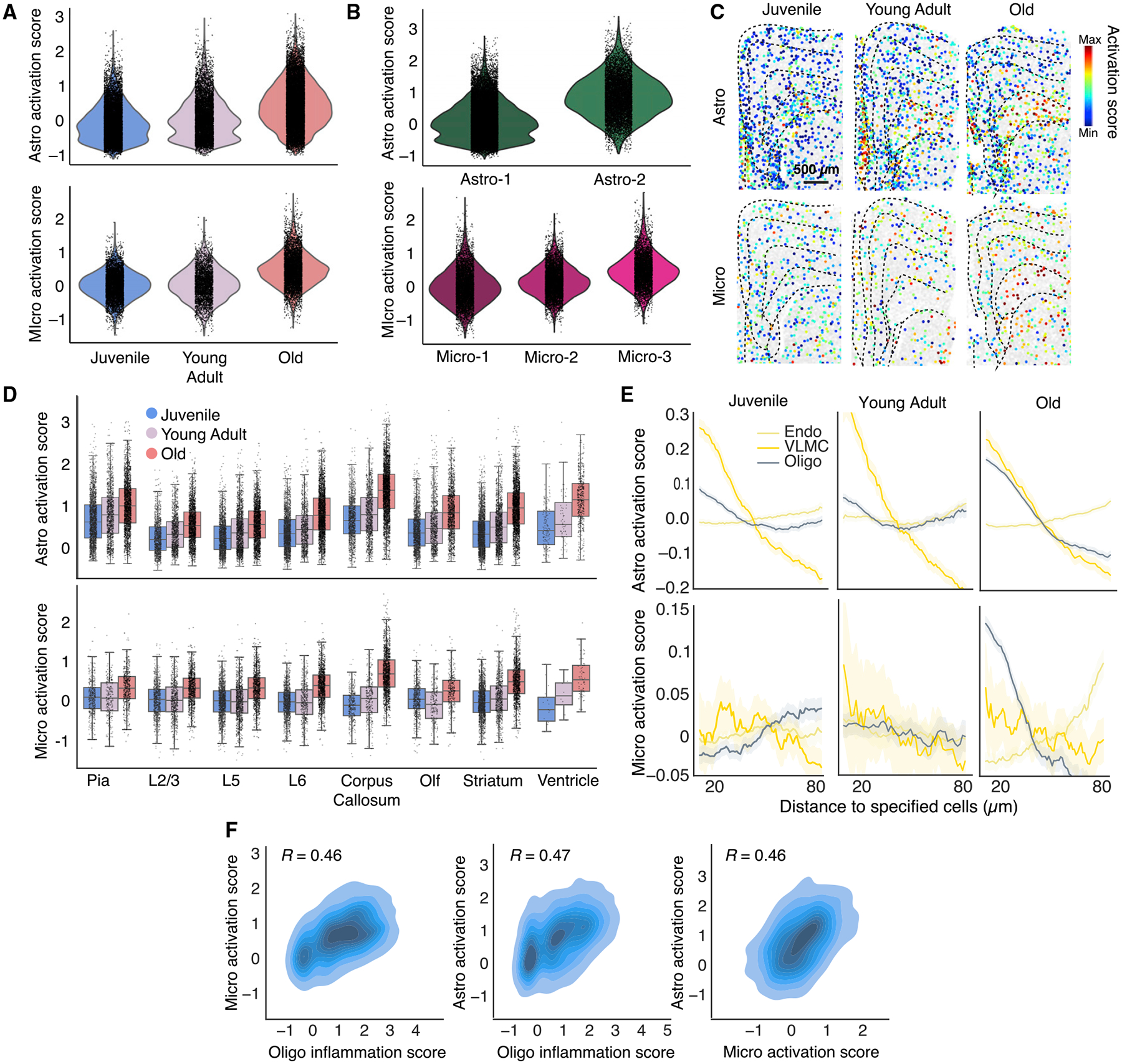
Spatially heterogeneous and cell-type-specific inflammatory activation signatures of brain aging (A) Activation scores of all astrocytes and microglia across the three different ages. Activation score is defined as the summed expression of a cell-type-specific subset of gene related to inflammatory activation, relative to background of randomly selected genes (STAR Methods). (B) Activation scores of specific astrocyte and microglia clusters. (C) Spatial maps of activation scores of astrocytes and microglia across the three different ages. Cells are colored by activation scores. Scale bar: 500 μm. (D) Per-cell activation scores of astrocytes and microglia in different anatomical regions across three ages. Data are presented as boxplots, with the box showing median and interquartile range and the whiskers showing minimum and maximum. (E) Average activation scores of astrocytes and microglia as a function of distance from neighboring oligodendrocytes, VLMCs, and endothelial cells across three ages. A constant that equals to the mean activation score across all distances is subtracted from each curve, and these constants for different curves are shown in STAR Methods. Data are presented as mean (solid line) ± SEM (shade). (F) (Left) correlation of activation scores of each microglial cell in corpus callosum with the average inflammation scores of oligodendrocytes within 30 μm of that microglial cell. (Middle) same as (left) but for astrocytes and oligodendrocytes. (Right) same as (left) but for astrocytes and microglia. Pearson correlation coefficients *R* are given. See also Figure S5 and Tables S1–S3.

To quantify the activation of these cell types and determine the spatial distributions of activated cells, we scored the activation levels of astrocytes and microglia imaged by MERFISH using genes previously shown to be specific for activated cells (STAR Methods). The activation scores for both astrocytes and microglia increased on average with age (Figure 5A). The astrocytic and microglial clusters enriched in old animals (Astro-2 and Micro-3) had higher activation scores than the other clusters of the same cell type (Figure 5B).

Notably, astrocytes and microglia exhibited distinct spatial signatures in their activation patterns. Astrocytes showed more pronounced spatial heterogeneity in activation, with the highest level of activation in the corpus callosum, as well as relatively strong activation near the ventricle and pia, but weaker activation in the cortex (Figures 5C and 5D). This spatial pattern was already apparent in juvenile and young adult animals and became more pronounced in old animals. Microglia activation, on the other hand, was more uniform across different regions, with hardly any spatial heterogeneity in juvenile and young adult animals (Figures 5C and 5D). As the animal aged, microglia activation increased more or less uniformly across different regions except for cells in the corpus callosum and near the ventricle, which showed a higher level of activation (Figures 5C and 5D).

Microglia and astrocytes may be activated by pro-inflammatory cytokines and chemokines that circulate in the blood^45^ or are released by brain-infiltrating immune cells,^15^ and we observed enrichment of macrophages near vascular cells in old animals (Figure S3). This observation prompted us to further examine whether the activation levels of astrocytes and microglia depended on their distance to vascular cells that separate the bloodstream (e.g., endothelial cells) or cerebrospinal fluid (e.g., VLMCs) from the interior of the brain. In addition, since we observed that several genes involved in inflammatory response and innate immune signaling (*Il18, Il33*, and *C4b*) were upregulated in oligodendrocytes (Figure 4B), we also examined the dependence of astrocyte and microglia activation on the distance to oligodendrocytes.

These dependencies were notably different between astrocytes and microglia. Astrocyte activation exhibited a strong dependence on the proximity to VLMCs in both young and old animals, and a dependence on the proximity to oligodendrocytes that increased substantially with age (Figure 5E). On the other hand, microglia did not show a substantial preference for activation near vascular cells, but aging-induced activation of microglia showed a strong dependence on their proximity to oligodendrocytes (Figure 5E). Moreover, we scored the inflammation level of oligodendrocytes using the expression levels of *Il33, Il18*, and *C4b* and observed that within the corpus callosum, the aging-induced activation levels of astrocytes and microglia were correlated with the inflammation level of nearby oligodendrocytes (Figure 5F), suggesting that the dependence on the proximity to oligodendrocytes was not simply a reflection of stronger activation of astrocytes and microglia in the corpus callosum but is likely related to the inflammatory response of oligodendrocytes over aging. The activation levels of astrocytes and microglia in the corpus callosum were also correlated with each other (Figure 5F).

The above results suggest multiple different mechanisms of non-neuronal cell activation, two of which showed strong spatial dependence: (1) activation of astrocytes near the surface of the vascular structures separating the cerebrospinal fluid and the brain, potentially caused by factors derived from cerebrospinal fluid, and (2) activation of microglia and astrocytes near oligodendrocytes in the corpus callosum. Only the second mechanism appeared to be aging specific. Consistent with the notion of two different activation mechanisms, the molecular signatures of activated astrocytes near the pia were different from those in the corpus callosum (Figure S5).

### Activation of glial and immune cells in response to systemic inflammatory challenge

The activation of astrocytes and microglia with age, reminiscent of brain inflammation, raises an interesting question as to how these age-related states compare with those induced by systemic inflammation. Peripheral administration of LPS is widely used to model brain inflammation associated with neurodegenerative disease.^46^ Although LPS itself is thought not to cross the blood-brain barrier, systemic release of cytokines and chemokines by peripheral immune cells upon LPS administration can broadly activate microglia and astrocytes throughout the brain.^25,47^

**Figure 6.**
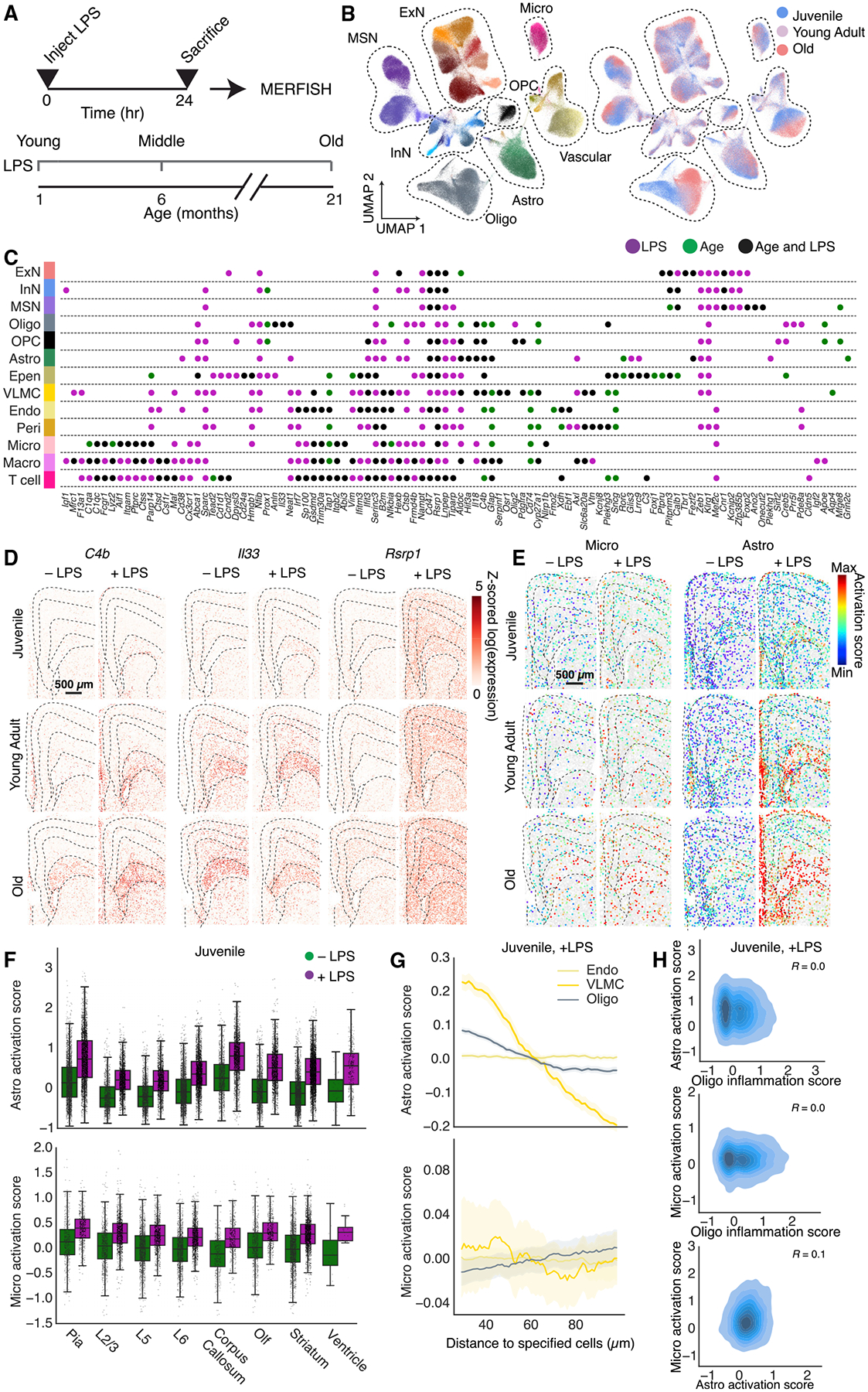
Gene expression changes and activations of cells in response to systemic inflammatory challenge by LPS treatment (A) Experimental scheme. (B) UMAP of cells colored by cell types (left) or ages (right) measured by MERFISH. (C) Genes substantially upregulated in response to LPS (magenta), age (green), or both LPS and age (black) for different cell types measured by MERFISH. Genes are considered substantially upregulated only if the change in Z-scored log(gene expression) is >2 with an FDR-adjusted p value <10^–4^. Only genes substantially upregulated in at least one condition for at least one cell type are shown. (D) Spatial maps of example genes that are upregulated with age and upon LPS treatment, showing the expression of the indicated gene across all cells within a section. Scale bar: 500 μm. (E) Spatial maps of activated microglia and astrocytes across the three different ages, with and without LPS treatment. Cells are colored by activation scores. Scale bar: 500 μm. (F) Per-cell activation scores for microglia and astrocytes in different anatomical regions in juvenile mice with LPS treatment. Boxplots are as defined in Figure 5D. (G) Activation scores of astrocytes and microglia as a function of distance from neighboring oligodendrocytes, VMLCs, and endothelial cells in juvenile mice after LPS treatment, as in Figure 5E. Data are presented as mean (solid line) ± SEM (shade). (H) Correlation of activation scores of microglia and astrocytes and inflammation score of oligodendrocytes, as in Figure 5F, in juvenile animals treated with LPS. See also Figures S6 and S7 and Tables S1–S3.

We injected mice at the three ages (~1 month, ~6 months, and ~21 months postnatal) with LPS (Figure 6A), euthanized the animals 24 h after LPS injection, and performed MERFISH measurements using our cell type and aging gene panels. ~350,000 cells passed quality control analysis, and we classified these cells by integrating the LPS dataset with the normal brain MERFISH dataset described earlier and transferred cell-type annotations without re-clustering (Figures 6B and S6A).

We observed a high degree of similarity between untreated and LPS-treated mice in terms of both the composition (Figures S6A–S6C) and the global spatial organization (Figure S6D) of the cell types. However, compared to untreated animals, LPS-treated animals showed a higher degree of enrichment of macrophages near vascular cells (Figure S6E), similar to that observed over the course of normal aging (Figure S3).

LPS induced substantial changes in the gene expression in a cell-type-specific manner, and some of the upregulated genes overlapped with the genes that showed age-related upregulation. To quantify these effects, we compared the extent to which specific genes were upregulated by LPS treatment and with age in the MERFISH data (Figure 6C and S6F). These analyses revealed both similarities and differences between LPS-induced changes and age-dependent changes. Many of the genes involved in innate immune response that were upregulated with age were also upregulated in responses to LPS (Figures 6C and S6F). There were, however, substantial quantitative variations in the relative extent of upregulation under the two conditions. For example, C4b was highly upregulated with age and further upregulated by LPS treatment, consistent with previous observations in astrocytes using bulk RNA-seq;^25^ *Il33* was strongly upregulated with age, whereas LPS treatment induced only very small additional upregulation of this gene; *Rsrp1* was more strongly upregulated in response to LPS (Figure 6D). There was also a subset of immune-response-related genes that were only substantially upregulated under one of the two conditions, according to the criteria that we used to define substantial upregulation (Figure 6C).

We also compared the activation patterns of astrocytes and microglia under LPS treatment and with those induced by aging. LPS increased the activation of astrocytes and microglia in animals at all ages (Figure 6E). Astrocytes were preferentially activated by LPS in the corpus callosum and near the pia and ventricle, whereas microglia were largely uniformly activated by LPS across all regions (Figures 6E, 6F, and S6G). Moreover, in juvenile and young adult animals, the activation of microglia by LPS did not depend on the proximity to oligodendrocytes or VLMCs, whereas the activation of astrocytes showed a strong dependence on the proximity to VLMCs and a weak dependence on the proximity to oligodendrocytes (Figures 6G, 6H, and S6H). In old animals, the dependence of microglia and astrocyte activation on the proximity to oligodendrocytes became even weaker in LPS treated animals than in untreated animals (compare Figure S6H to Figure 5E), potentially because the distance dependence of the age-related activation is masked to some extent by the distance-independent activation by LPS. We note that although the untreated and LPS-treated mice were part of separate animal cohorts, the cohort-to-cohort variation was substantially smaller than the differences observed between +LPS and −LPS conditions (Figure S7).

Taken together, these results showed interesting commonality and differences between age- and LPS-induced activations of non-neuronal cells: while both conditions induced spatially heterogeneous activation of astrocytes with enrichment near the cerebrospinal-fluid-brain barriers and dispersed activation of microglia, aging uniquely induced microglia activation, and potentially related increase in astrocyte activation, near oligodendrocytes in the corpus callosum.

## DISCUSSION

Many hypotheses have been suggested for the causes of brain function decline with age, ranging from changes in synaptic connectivity or physiology^2^ to senescence of glial and immune cells and the role of circulating inflammatory factors.^48^ Previous transcriptomic studies have revealed widespread changes in cell state in specific cell types with age, with many of these studies highlighting a role for increased inflammation as a key aspect of brain aging.^17–20,25–30^ However, to understand how these changes may impact specific brain functions and to gain insights into the mechanisms underlying age-related functional decline, it is crucial to characterize both the molecular and cellular signatures and the spatial locations of these changes within the brain.

Here, we used spatially resolved single-cell transcriptomics to systemically uncover changes in the molecular signatures and spatial organizations of brain cells in the mouse frontal cortex and striatum over the animal’s lifespan. By integrating snRNA-seq and MERFISH data, we generated a spatially resolved cell atlas of the aging brain with a genome-wide expression profile associated with each cell. We observed more pronounced, and qualitatively different, age-induced changes in non-neuronal cells compared to neurons, and these changes in non-neuronal cell exhibited specific spatial patterns.

At the molecular level, many of the genes upregulated with age in non-neuronal cells were related to activation of inflammatory pathways associated with innate immunity, while neuronal cell populations displayed different transcriptional changes, many of which related to neurodegenerative diseases, oxidative stress, and mitochondria functions. Immune cells and secreted factors such as cytokines are widely involved in the maintenance of tissue homeostasis.^7^ Hence, the observed upregulation of genes related to inflammation and innate immunity in immune and glial cells within the brain could be an indication of dysregulated tissue homeostasis that may broadly affect the function of the nervous system.

While the spatial organization of neurons were largely stable with age, we observed notable spatially dependent changes in the cell states of non-neuronal cells, with specific oligodendrocyte and astrocyte states emerging in the corpus callosum of the aging brain. Interestingly, inflammatory activation of microglia and astrocytes during aging showed distinct spatial patterns: both cell types exhibited the strongest activation in the corpus callosum, a location that also showed strong inflammatory changes of oligodendrocytes, whereas astrocytes also showed increased activation near the pia. Overall, astrocyte activation appeared to be more spatially heterogeneous than microglia activation. Taken together, these results highlight the white matter of the corpus callosum as a hotspot of age-associated inflammatory changes in the brain.

Previous MRI studies in humans have revealed that prefrontal white matter is highly susceptible to age-related reduction in volume,^49^ and that the degree of white matter changes are associated with cognitive decline.^50^ Electron microscopy studies of non-human primate brain aging have revealed major alterations specifically in the white matter, particularly in the disruption of the myelin sheath.^51^ White-matter microglia reactivity has also been related to aging^52^ and diseases.^53^ Expanding upon these findings, our results suggest that changes in the oligodendrocytes and myelinated axons, and their associated microglial and astrocytic reactivity in the white matter, may be an important factor in age-associated cognitive deficits. Our observations that the activation levels of microglia and astrocytes in the corpus callosum are correlated with each other and with the inflammation level of oligodendrocytes marked by cytokines like *Il33* further suggest potential molecular mechanisms underlying this inflammatory activation. In one scenario, the elevated expression of pro-inflammatory cytokine *Il33* in aged oligodendrocytes may activate microglia through the *Il33* receptor, which is known to be expressed in microglia.^54^ In a second and potentially related model, excessive myelin degradation can induce activation of phagocytosing microglia that become overloaded with myelin fragments.^52,55^ In both scenarios, activated microglia can in turn activate astrocytes through secretion of pro-inflammatory cytokines and complement proteins.^40^ Activated astrocytes and microglia may in turn exacerbate oligodendrocyte and myelin degeneration.^40,56^

Our result further showed that microglia and astrocyte activation induced by aging exhibited both similarities to and notable differences from those induced by systemic inflammatory challenge. On the one hand, many of the same genes were upregulated both by acute LPS treatment and during aging, consistent with previous observations of similar brain responses to aging and LPS treatment in terms of bulk expression of pro-inflammatory cytokines and markers of activated states of those cell types.^25,57–60^ On the other hand, we also observed differences in cell-state changes induced by aging and LPS treatment in both gene-expression and spatial patterns. In particular, activation of microglia and astrocytes associated with the inflammation of oligodendrocytes in white matter of the corpus callosum was uniquely observed in the aging brain. Identifying the molecular mechanisms underlying these commonalities and differences will require further mechanistic investigation of the roles of specific cytokines and other signaling pathways in the brain. Indeed, these two processes may intersect: intrinsic aging-related degenerative processes within the brain, which locally disrupt tissue homeostasis, may prime cells into a pro-inflammatory state, which could then be exacerbated by systemic factors.^45^

The functional consequences of the disruptions to non-neuronal cellular homeostasis on neural circuits remain to be investigated. Many of the genes that we observed to be upregulated during aging, such as interleukins and complement proteins, have been shown to play a crucial role in regulating neural circuit organization and function via interactions between neurons and non-neuronal cells during development.^54,61^ Our cell atlas of the aging brain could facilitate future studies aiming to determine whether spatially localized upregulation of these molecules with age in turn causes localized disruptions to neural circuit function. Integrating these studies in mice with spatial transcriptomic measurements in humans in multiple conditions (normal aging, acute brain injuries, as well as neurodegenerative disorders) may reveal how the inflammatory activation of non-neuronal cells contributes to cognitive impairment associated with advanced age and diseases at the neuronal and circuit levels.

### Limitations of the study

Because we did not perform snRNA-seq measurements on LPS-treated mice, our comparison of the effects of normal aging and acute inflammatory challenge by LPS was limited to the genes measured in our MERFISH panel. In addition, we only measured female mice, and future comparison between female and male mice could capture sex-specific changes in brain aging. Finally, this study was performed only on the frontal cortex and striatum. Extension of the study to the whole brain may capture a more complete picture of brain aging and identify differences between brain regions.

## STAR★METHODS

### RESOURCE AVAILABILITY

#### Lead contact

Further information and requests for resources and reagents should be directed to and will be fulfilled by the lead contact, Xiaowei Zhuang (zhuang@chemistry.harvard.edu).

#### Materials availability

Oligonucleotide encoding probe sequences used for MERFISH imaging can be found in Table S2. Oligonucleotide readout probe sequences used for MERFISH imaging can be found in Table S3. These probes or templates for making these probes can be purchased from commercial sources, as described in the Key resources table.

#### Data and code availability

- snRNA-seq data reported in this work are available at NCBI GEO data repository (GSE207848). MERFISH and snRNA-seq data reported in this work are available at CELL x GENE repository (https://cellxgene.cziscience.com/collections/31937775-0602-4e52-a799-b6acdd2bac2e).
- All original analysis code generated in this work is available at: https://github.com/ZhuangLab/SpatialBrainAgingCell22.
- Any additional information required to reanalyze the data reported in this paper is available from the lead contact upon request.

### EXPERIMENTAL MODEL AND SUBJECT DETAILS

#### Animals

Female C57BL6/J mice were used in this study. Mice were obtained from Jackson Laboratory at an age one week younger than the target age for sacrifice [~1-month (4-week), ~6-month (24-week), and ~21-month (90-week) postnatal], and then housed at Harvard University Animal Facility for 1 week to acclimate before sacrifice. Mice were maintained on a 12 h light/12 h dark cycle (14:00 to 02:00 dark period) at a temperature of 22 ± 1°C, a humidity of 30–70%, with *ad libitum* access to food and water. Animal care and experiments were carried out in accordance with NIH guidelines and were approved by the Harvard University Institutional Animal Care and Use Committee (IACUC).

### METHOD DETAILS

#### Single-nucleus RNA-sequencing

Female mice aged 4 weeks or 90 weeks old were anesthetized with isofluorane and then acutely decapitated. Their brains were quickly harvested and cut into hemispheres and each hemisphere was frozen immediately on dry ice Optimal Cutting Temperature Compound (OCT, Fisher HealthCare) and then stored at −80°C until sectioning. Brains were taken from storage at −80°C and warmed to −18°C in a cryostat (Leica) for 20 min before sectioning. Sections were discarded until the beginning of frontal cortex was apparent. Brains were then blocked on the cryostat using a razor blade to a region containing frontal cortex and striatum. 100 μm coronal sections were then collected approximately from A/P +2 mm to A/P +1 mm, relative to bregma. The resulting sections were collected in an Eppendorft tube and stored at −80°C until snRNA-seq library preparation.

For snRNA-seq library preparation, nuclei were dounced in Nuclei EZ Prep nuclei extraction buffer (Sigma) + 1% Rnase Inhibitor. Nuclei were then spun down, filtered through a 70 μm filter, stained with DAPI, and sorted on a FACS machine (BD FacsAria) to separate nuclei from debris. The resulting clean nuclei preparation were then counted and encapsulated on a 10X Genomics Chromium machine, using the 3’ Transcriptome V3.1 kit (10X Genomics). After encapsulation, the resulting libraries were reverse transcribed, amplified as cDNA, fragmented, and amplified as a final library following the manufacturer’s instructions. The resulting libraries were sequenced on a NovaSeq S4 flowcell (Illumina) to a target depth of ~50,000 reads per nucleus.

#### snRNA-seq data analysis

Raw reads were mapped to the mm10 mouse reference genome and demultiplexed to generate a per-cell count matrix using CellRanger pipeline (10X Genomics). The resulting data were analyzed in Python using standard methods implemented in the package Scanpy. Briefly, putative doublets were first removed using Scrublet.^63^ Cells with <2,500 UMIs per cell and <1,000 genes per cell were removed. Genes detected in <3 cells were removed. Following standard procedures in Scanpy, per-cell counts were normalized to sum to 10^4^ counts per cells and log-transformed. A multi-level clustering approach was taken, where the cells were first clustered into major cell types then into clusters within those cell types as described in Figure S1A. Briefly, at each level highly variable genes were determined and included in the per-cell expression matrix, the total UMI number per cell and expression of mitochondrial genes were regressed out, and the resulting residuals were Z-scored. Principal component analysis was used to reduce the dimensionality to 50 principal components. A nearest neighbor graph was computed between cells using these principal components, and Leiden clustering was applied to separate the cells into distinct clusters.

First all cells were clustered into neurons and non-neuronal cells. Within the neurons, cells were clustered into inhibitory and excitatory neurons. Inhibitory neurons were further subclustered into medium spiny neurons (MSNs) and non-MSNs. Non-neuronal cells were subclustered into astrocytes, microglia, macrophages, oligodendrocytes, pericytes, vascular leptomeningeal cells (VLMCs). Each major cell type (excitatory, inhibitory, MSN, astrocytes, microglia, macrophages oligodendrocytes, pericytes, VLMCs) was then subclustered to obtain the final list of cell clusters.

#### Gene selection for MERFISH measurements

Genes were selected for MERFISH using a combination of automated and manual approaches. First, to identify age-related genes, linear regression was used to identify genes that were differentially expressed between two different ages (4-week and 90-week postnatal) in individual cell types or clusters determined by snRNA-seq. Briefly, using statsmodels, a Generalized Linear Model with a Negative Binomial link function was fit to the log-transformed UMI counts per cell for each gene *y*_*i*_:

$$y_{i}\sim C(\text{age})+{\text{log}}_{10}(\text{total}\_\text{counts})+\text{intercept}+\varepsilon$$

where C(*age*) is a binary categorical variable with the 4-week value set to be the reference level (i.e. C(4-week) = 0) and the C(90-week) value determined from the model. This model was fit separately for each cell type or cluster, which was compared with a null model that only accounts for technical variation in the total molecule counts per cell:

$$y_{i}\sim {\text{log}}_{10}(\text{total}\_\text{counts})+\text{intercept}+\varepsilon$$

A likelihood ratio test was then computed between the full and reduced models to determine a p-value. These p-values were corrected for multiple hypothesis testing across all genes in all cell types or clusters to give the FDR-adjusted p-values, and genes with an FDR-adjusted p-value <10^–6^ were considered. For each cell type and cluster, the genes differentially expressed between the two ages were sorted by the fitted C(90-week) value, and the top N genes with at least C(90-week) > 1.5 were included in the aging gene panel. For each major cell type, we included 5 genes and for each fine-leaflet cell cluster we included 2 genes. This approach attempts to balance the gene panel across all cell types and clusters, even if certain cell types or clusters may have more or fewer total numbers of differentially expressed genes with age.

To identify cell-type-marker genes, marker genes were identified for a particular cell population (cell type or cluster) using a one-vs-all approach. For each cell population, a t-test was performed for each gene between the cells within the cell population and all other cells not in that population. The resulting p-values were corrected for multiple hypothesis testing to give FDR-adjusted p-value. A gene was identified as a cell-type marker for a certain cell population if it satisfy the following conditions: i) it was expressed in at least 40% of cells within the specified cell population, ii) it had an FDR-adjusted p-value <0.05, iii) it had a gene expression in the specified cell population that was at least 2-fold higher than the average expression in all cells not in that population, and iv) it was expressed in a fraction of cells within the specified cell population that was at least three times higher than the fraction of cells not in this population that expression the gene. Finally, the marker genes for each cell population were sorted by fold change in expression relative to the cell outside the cell population, and the top 15 marker genes for each cell population were then saved and used for marker selection. To select the final set of markers, we greedily added marker genes to the list such that each cell type or cluster had at least two marker genes included in the final gene panel.

In addition to these markers, known cortical layer markers,^36^ genes related to microglial^16,24,43^ and astrocyte^25,40^ activation, broad transcriptomic markers of aging,^18^ and markers for various immune cell types (T cells, B cells, macrophages)^7,68,69^ were curated from the literature and included. In total, 212 genes were included in the cell-type-marker gene panel and 204 genes were included in the aging gene panel.

#### Design and construction of MERFISH encoding probes

After the aging and cell-type-marker MERFISH gene panels were selected, a 20-bit code was created for the gene panels (Table S1). Briefly, a 20-bit Hamming-weight 4 code was generated by first listing all possible combinations of 4 “on” bits embedded within 20 bits. This list was shuffled, and the first bit combination was randomly selected as the initial barcode. Additional barcodes were then added from this list by iterating through the other randomly shuffled barcodes and greedily adding to the codebook each additional barcode that had a Hamming Distance of at least 4 from all barcodes currently in the codebook. A collection of 500 such randomly sampled codes was generated, and each was scored on the total number of barcodes and the variance of number of barcodes utilizing each bit. A 20-bit, Hamming Distance 4 and Hamming-weight 4 code with the lowest variance that included at least 200 codewords was then used for both libraries, resulting in a 223-codeword final codebook.

Next, individual genes were assigned to barcodes in the codebooks. This assignment was initially random, then optimized to increase the average expected uniformity of the density of molecules per cell that were detected in each bit. This optimization was performed iteratively, using a simulated annealing algorithm to maximize the uniformity of expression across bits on average across all cell types.

First, we estimated the expected total number of molecules per cell for each bit as the sum of the expression (determined by snRNA-seq) of genes with barcode reading “1” at that bit. This was computed for each cell type, and the weighted average across cell types was computed weighted by the cell abundance in individual cell types. Individual gene assignments to barcodes were then swapped, and the average expression per cell per bit was re-computed. Assignment swaps that decreased the variance across bits were kept. This process was repeated until the algorithm converged when the variance stopped decreasing.

For each gene, we then designed a total of 92 encoding probes targeting that gene’s mRNA sequence (Table S2). The encoding probes were designed as previously described.^70^ Briefly, we selected regions with GC content between 30 and 70%, melting temperature T_m_ within 60–80°C, isoform specificity index between 0.75 and 1, gene specificity index between 0.75 and 1, and no homology longer than 15 nt to rRNAs or tRNAs. For each library, each of the 20 bits was assigned to a 20-nt three-letter (A, T, C) readout sequence. Each encoding probe was constructed by concatenating the 30-nt target region with three 20-nt readout sequences for each probe. The readout sequences for each gene were randomly shuffled across all 92 encoding probes for that gene. The encoding probes additionally contained a 20-nt reverse transcription primer sequence at the 5’ end and a T7 promoter at the 3’ end, which also functioned as PCR primer sequences.

#### MERFISH encoding probe library and readout probe preparation

The encoding probe library was synthesized using large-scale arrayed oligo synthesis (Twist Biosciences) and then amplified, as previously described.^70^ Briefly, the initial library was amplified using limited cycle PCR (Phusion Polymerase, NEB) monitored via qPCR. The library was then converted to RNA via *in vitro* T7 transcription (HiScribe T7 Quick High Yield Kit, New England Biolabs) from a T7 promoter integrated into the PCR product. The resulting RNA product was purified (RNA Clean and Concentrate, Zymo Research) and reverse transcribed (Maxima H– Reverse Transcriptase, ThermoFisher). The RNA in the resulting RNA:DNA hybrid was degraded using alkaline hydrolysis, and the final ssDNA product was first desalted via buffer exchange through a 7K MWCO desalting column (ThermoFisher) then concentrated using a phenol:chloroform extraction and ethanol precipitation, resulting in a 5–10 nM/probe final library.

For the 416-gene panel used in this study, 40 readout probes (Table S3) were used, each complementary to one of the 40 readout sequences on the encoding probes. For readout, the first twenty readout probes correspond to the 20 bits of the code used for the cell-type-marker gene panel and the remaining twenty readout probes correspond to the 20 bits of the code used for the aging-related gene panel. Each readout probe was conjugated to one of the two dye molecule (Alexa Fluor 750 or Cy5) via a disulfide link-age, as previously described.^70^ The readout probes were obtained from Integrated DNA Technologies and resuspended immediately in Tris-EDTA (TE) buffer, pH 8 (Thermo Fisher), to a concentration of 100 μM, and stored at −20°C until use.

#### Tissue sample preparation for MERFISH

Brains were prepared as described for snRNA-seq, with the addition of mice at the age of ~6-month (24-week) postnatal without LPS treatment, and mice at the ages of ~1-month (4-week), ~6-month (24-week), and ~21-month (90-week) postnatal following LPS injection. Sectioning was performed on a cryostat at −18°C. slices were removed and discarded until the frontal cortex and striatal target region was reached. In order to capture comparable sections across animals, starting from approximately A/P +2 mm relative to bregma, every other 10 μm section was captured onto a set of 6–8 lysine-coat silanized coverslips for MERFISH imaging, with each coverslip ultimately containing 3 to 4 individual sections. The coverslips were cleaned, silanized, and treated with poly-lysine as previously described.^36^ The same anatomical region was identified for imaging post hoc after sample preparation, before the start of MERFISH imaging.

Tissue sections were fixed in 4% paraformaldehyde (Electron Microscopy Sciences) for 20 min, washed three times in 1 × D-PBS, and then stored in 70% EtOH (Koptec) at 4°C for at least 18 h to permeabilize the tissue. Tissue slices from the same mouse were cut at the same time and distributed onto 6–8 coverslips; multiple mice were sectioned at the same time. Coverslips were stored in 70% EtOH for less than two weeks until each biological replicate was successfully imaged once.

The tissue sections were stained with MERFISH encoding probes as previously described.^71^ Briefly, the samples were removed from 70% EtOH and washed with 2× saline sodium citrate (SSC) three times. We then removed excess 2× SSC by blotting with a Kimwipe and inverted the coverslip onto a 50 μL droplet of encoding probe mixture in a Parafilm-coated Petri dish. The encoding probe mixture contained approximately 1 nM of each encoding probe, 1 μM of polyA-anchor probe (Integrated DNA Technologies) in 2× SSC with 30% v/v formamide, 0.1% wt/v yeast tRNA (ThermoFisher), 10% v/v dextran sulfate (Sigma), and 1% v/v murine Rnase inhibitor (New England Biolabs). The polyA-anchor probe containing a mixture of DNA and LNA nucleotides (/5Acryd/TTGAGTGGATGGAGTGTAATT+TT+TT + TT + TT + TT + TT + TT + TT + TT + T, where T+ is locked nucleic acid, and/5Acryd/is 5’ acrydite modification) was hybridized to the polyA sequence on the polyadenylated mRNAs, allowing these RNAs to be anchored to a polyacrylamide gel as described below. The sample was then incubated for 36–48 h at 37°C.

After hybridization, the samples were washed in 2× SSC with 30% v/v formamide for 30 min at 47°C for a total of two times to remove excess encoding probes and polyA-anchor probes. Tissue samples were cleared to remove lipids and proteins that contribute fluorescence background, as previously described.^71^ Briefly, the samples were embedded in a thin 4% polyacrylamide gel and were then treated with a digestion buffer of 2% v/v SDS(Thermo Fisher), 0.5% v/v Triton X-100 (Sigma) and 1% v/v proteinase K (New England Biolabs) in 2× SSC for 36–48 h at 37°C. After digestion, the coverslips were washed in 2× SSC for 30 min for a total of four washes and then stored at 4°C in storage buffer of 2× SSC, 1% v/v murine Rnase inhibitor (New England Biolabs) before imaging.

### MERFISH IMAGING

We used a custom-built imaging setup in this study as previously described.^62^ All buffers and readout-probe mixtures were flowed onto the sample using a home-built, automatic fluidics system as previously described.^62^ Briefly, the samples were stained with 1 μg/mL Hoechst 33,342 (ThermoFisher) and then loaded into a commercial flow chamber (Bioptechs) with a 0.75-mm-thick flow gasket. The first MERFISH round, containing both the first two readout probes labeled with Cy5 and Alexa Fluor 750, as well as a probe complementary to the polyA anchor labeled with Alexa Fluor 488, were then stained on the microscope. For each hybridization round, the fluorescent probes were hybridized in a buffer containing 2× SSC, 10% v/v ethylene carbonate (Sigma), and 0.1% Triton X-100, and were diluted to a final concentration of 3 nM. The samples were stained for 15 min, and then washed with readout probe buffer. Finally, imaging buffer was flowed into the chamber. The imaging buffer consisted of 2× SSC, 10% w/v glucose (Sigma), 60 mM Tris-HCl pH8.0, ~0.5 mg/mL glucose oxidase (Sigma), 0.05 mg/mL catalase (Sigma) 50 μM trolox quinone (generated by UV irradiation of Trolox, 0.5 mg/mL 6-hydroxy-2,5,7,8-tetramethylchroman-2-carboxylic acid (Trolox, Sigma), and 0.2% v/v murine RNAase inhibitor (New England Biolabs).

After the readouts for the first round were hybridized, the sample was imaging with a low magnification objective (CFI Plan Apo Lambda ×10, Nikon) with 405-nm illumination to produce a low-resolution mosaic of the sections in the Hoeschst channel. We used this mosaic to generate a grid of tiled fields-of-view (FOV) covering the relevant areas of frontal cortex and striatum. We then switched to a high-magnification, high-numerical aperture objective (CFI Plan Apo Lambda ×60, Nikon), and imaged each FOV with a 7-plane z stack with 1.5 μm spacing between the adjacent z-planes to cover the entire 10 μm thickness of the tissue section. For each FOV, we took images in the 750-nm, 650-nm, 560-nm, 488-nm and 405-nm channels: one image of the orange fiducial beads (560-nm) at the bottom z-plane, which was used as a fiducial marker to register the position of each FOV across multiple rounds of hybridization. For each z-plane, we took images of the readout probes (Alexa Fluor 750 and Cy5, 750-nm and 650-nm respectively), polyA probes (488-nm), and Hoeschst nuclear DNA stain (405-nm).

After the first round of imaging, the dyes were cleaved from the readout probes by flowing 2.5 mL of cleavage buffer (2× SSC and 50 mM of Tris (2-carboxyethyl) phosphine [GoldBio]) and incubating for 15 min, which cleaved the disulfide bond linking the dye to the readout oligonucleotide. The excess TCEP was removed by washing with 1.5 mL of 2× SSC.

For subsequent rounds of imaging, the same steps were carried out using readout-probe mixture containing 3 nM of the appropriate Alexa Fluor 750- and Cy5-labeled readout probes for each round.

The two gene panels (cell-type-marker panel and age-related gene panel) were imaged back-to-back on the same tissue sections, each gene panel was imaged in 10 rounds with two readout probes per round to readout the 20 bits. Each experiment took approximately 24–36 h to image the relevant fields of view from 2–4 coronal slices.

#### MERFISH data processing

Imaging data were uploaded to the Harvard FAS Research Computing cluster and decoded using our previously published MERlin pipeline^62^ with modifications on cell segmentation as described below. This pipeline provides the gene identity and spatial coordinates of each decoded molecules. For cell segmentation, we used the ‘cyto2’ model CellPose,^67^ a deep learning based cell segmentation algorithm. This was applied only to Hoeschst-stained nuclei in order to avoid incorrect segmentation of neighboring cells. Decoding molecules were then assigned to the segmented nuclei to produce a cell-by-gene matrix that list the number of molecules decoded for each gene in each cell.

The cell-type-marker gene panel and aging-related gene panel were decoded separately. The segmented nuclei determined in these two decoding runs were then aligned by identifying the mutual nearest neighbor nuclei that were within 5 μm of each other. The very small number of nuclei that did not have a mutual nearest neighbor within this distance cutoff were removed from the dataset, as potentially incorrectly segmented cells, and the remaining nuclei were each assigned a vector of gene expression counts that included decoding results from both decoding runs. During data analysis, we observed that the readout of bit #20 of the aging-related gene panel was consistently dim in the majority of experiments, suggesting that genes detected in this bit may have a reduced detection efficiency. We thus excluded from all subsequent analysis the 40 genes that should be detected in this bit (i.e. genes whose barcodes read “1” at this bit), although the major conclusions in this paper were not altered if we included these genes in the subsequent analysis.

After decoding, cells from all MERFISH experiments were combined into a single dataset. Putative doublets were removed using Scrublet. Cells were then filtered to remove all cells with <20 molecule counts per cell or with <5 genes detected per cell. Cells that had a volume <100 μm^3^ or a volume >3 times the median volume across all cells were removed. Each cell’s gene expression values were normalized by dividing by the volume of that cell. The total normalized gene expression was computed for each cell, and cells with total normalized expression in the top and bottom 2% quantile were removed. Finally, these normalized values were scaled such that the sum of gene expression values per cell was equal to 250. The gene expression values were then log-transformed and Z-scored.

#### Integrated clustering analysis of the MERFISH and snRNA-seq data

The MERFISH expression matrix was concatenated with the normalized, log-transformed, and *Z* score snRNA-seq expression matrix, which was subsetted to include only the genes in the MERFISH gene panels. These data were then subjected to a two-step data integration and batch correction process, to first correct for modality-specific bias then for batch-specific bias using Harmony^35^ and BBKNN^65^ respectively. First, Principal component analysis was performed on the join data matrix. Harmony was then used to adjust the principal components for modality-specific (MERFISH or snRNA-seq) effects, producing an integrated representation in the principal component space. Second, these integrated principal components were then used by BBKNN to compute a batch-corrected nearest neighbor graph, where each batch was an experimental run of MERFISH or snRNA-seq. This batch corrected nearest neighbor graph was subsequently used to further reduce the dimensionality of the dataset via UMAP or to compute integrated clusters via Leiden clustering. For comparison of alternative integration methods, we also used Allcools^38^ instead of Harmony to correct for modality-specific bias.

To arrive at the final set of clusters, a semi-automated multi-level clustering approach was performed. Similar to the clustering approach used for snRNA-seq alone, cells were first clustered into neurons and non-neuronal cells. The neurons were then subclustered into excitatory and inhibitory neurons, and the inhibitory neurons were subclustered into MSNs and non-MSNs. The non-neuronal cells were clustered into oligodendrocytes, OPCs, astrocytes, microglia, vascular cells, and immune cells. These major cell types were then subclustered to arrive at the final list of clusters. Briefly, for each major cell type, the Harmony-corrected principal components were used via BBKNN to compute a batch-corrected nearest neighbor graph. This nearest neighbor graph was then used to perform Leiden clustering and to compute a UMAP plot for each major cell type. For each major cell type, differential gene expression was computed between each pair of subclusters using a *t*-test and the spatial locations of each cluster were plotted for manual inspection. The few clusters that appeared over-segmented based on heuristic criteria (no unique differentially expressed genes between the two clusters, largely overlap in UMAP space between the two clusters, a cluster with a very small cell number intermingled with a cluster with larger cell number in UMAP space) were then manually merged. The final set of clusters were annotated based on comparison of their key marker genes and/or spatial locations with previously annotated datasets.^36,37,72,73^

#### Imputation of gene expression

To impute the genome-wide expression profiles of the cells measured by MERFISH, the gene expression profiles of the snRNA-seq cells most similar to each cell measured with MERFISH were averaged together. This computation used the PCA embedding produced through Harmony or Allcools integration to identify the nearest neighboring snRNA-seq cells for each MERFISH cell, using the top 30 principal components in the jointly embedded PCA decomposition. The expression profiles of the 10 nearest neighboring snRNA-seq cells for each MERFISH cell was averaged together to produce the imputed gene expression profile for that MERFISH cell. To prevent the size of the dataset from exceeding available computer RAM, this imputation process was run on a subset of ~7,400 genes that included all highly-variable genes in neurons and non-neuronal cells in the snRNA-seq dataset, all genes measured by MERFISH, and all genes differentially expressed with age at the cell type and cluster level.

To assess the correlation between imputed results and results measured by MERFISH, we computed two different sets of correlation coefficients. In the first set, we calculated the Pearson correlation coefficient between imputed and measured spatial maps for each gene. For this purpose, the expression values of individual genes measured by MERFISH or imputed from snRNA-seq/MERFISH integration were binned at 150-μm resolution by computing the mean expression of that gene in all cells within a bin, in each tissue section separately. The Pearson correlation was then computed across all bins between the measured and imputed values for each specific gene. In the second set, we calculated the Pearson correlation coefficient between imputed and measured gene expression profiles for each cell type within each anatomic region. For this purpose, we identified all cells of a particular cell type within a particular anatomic region, determined the average Z-scored MERFISH gene expression value and average imputed gene expression value for each gene across those cells, and then computed the Pearson correlation between measured and imputed average gene expression profiles.

#### LPS injection experiment

Female C57BL6/J mice were injected intraperitoneally with 0.5 mg/kg lipopolysaccharide (LPS) derived from *Escherichia coli* O 111:B4 (Sigma) diluted in PBS at Zeitgeiber Time 9. Animals were euthanized 24 h after injection and brains harvested for MERFISH analysis. LPS was titrated following reconstitution to optimize dosage and ensure consistent potency across experiments.

### QUANTIFICATION AND STATISTICAL ANALYSIS

#### Brain region segmentation

In order to automatically segment anatomical regions across the many individual sections included in the MERFISH experiment, we developed a semi-supervised method to cluster cells based on the cell type composition of their local spatial neighborhood. For each cell, we computed the abundance of cells from all clusters within a 100-μm radius of this cell, presented in the form of a vector with N_cluster_ dimensions, where N_cluster_ in the number of clusters. We then combined these vectors across all cells to form an N_cell_ x N_cluster_ matrix, where N_cell_ is the number of cells. We applied principal components analysis to this matrix to reduce the dimensionality of this matrix, and then applied *k*-means clustering to segment the cells into *k* = 20 spatial clusters. This produced an overclustered segmentation of cells in space. We then hierarchically ordered these spatial clusters and manually merged spatial clusters that were near each other in the cluster hierarchy and appeared over-segmented when their spatial profiles were plotted, to arrive at a final set of 8 spatial clusters, which map closely to known anatomical structures, including the pia, cortical layer 2/3 (L), cortical layer 5 (L5), cortical layer 6 (L6), corpus callosum, striatum, ventricle, and subcortical olfactory areas.

#### Gene module analysis

Gene modules were identified from scRNA-seq data by first grouping cells from each major cell type. For each group, variable genes were then selected and a gene-gene correlation matrix was computed by taking the first 50 singular values from the singular value decomposition of the gene expression matrix and computing the dot product of it with its transpose. This gene-gene correlation matrix was then Z-scored and Z-scores less than 0.1 were set to zero to sparsify the matrix. Each gene in this matrix was then reduced to 2 dimensions using UMAP. Genes were then clustered into modules in this reduced dimensional space using the DBSCAN clustering algorithm. To remove modules that were not associated with any genes in a statistically significant manner, we compared the mean gene-gene correlation per module under this clustering with a shuffled distribution where the module identities of the genes were randomly permuted 1000 times to determine the p-value. These p-values were then FDR corrected and any modules with FDR <0.1 were removed.

#### Aging-related analysis

Activation scores were computed from the normalized, log-transformed, Z-scored gene expression values using the score_genes function in Scanpy, which computes the summed expression value of a set of genes minus the average expression of randomly selected background genes. The genes used for astrocyte activation were: *C4b, C3, Serpina3n, Cxcl10, Gfap, Vim, Il18*, and *Hif3a*.^25,40,74^ The genes used for microglial activation were: *B2m, Trem2, Ccl2, Apoe, Axl, Itgax, Cd9, C1qa, C1qc, Lyz2, Ctss*^16,24,43^. The genes used for calculating oligodendrocyte inflammation score were: *C4b, Il33*, and *Il18*. These inflammation-and innate-immunity-related genes showed differential expression in oligodendrocytes over aging.

To compute the activation scores of microglia or astrocytes as a function of distance to another cell type, the average activation scores of all microglia or astrocytes were computed as a function of distance from a reference cell type (Oligo, Endo, or VLMC). For each individual astrocyte or microglial cell, the nearest neighbor of a particular comparison cell type was identified within a radius of 80 μm of that astrocyte or microglia using a *k*D-tree search implemented in scikit-learn. The distance from the astrocyte or microglia to that comparison cell type was saved along with that astrocyte or microglia’s activation score. The average activation score for all microglia or astrocytes were computed at 1 μm stepping from 0 to 80 μm, with a sliding 30-μm-wide window. Finally, the mean activation across this range was subtracted from each curve of the astrocyte/microglia activation score as a function of distance to a specific cell type. The subtracted mean activation scores for each condition are listed below:

Astrocyte activation:

Distance to endothelial cell dependence (Juvenile, −LPS: −0.01; Juvenile, +LPS: 0.47; Young adult, −LPS: 0.07; Young adult, +LPS: 0.69; Old, −LPS: 0.40; Old, +LPS: 1.05).

Distance to VLMC dependence (Juvenile, −LPS: 0.26; Juvenile, +LPS: 0.86; Young adult, −LPS: 0.37; Young adult, +LPS: 1.15; Old, −LPS: 0.65; Old, +LPS: 1.46).

Distance to oligodendrocyte dependence (Juvenile, −LPS: −0.03; Juvenile, +LPS: 0.46; Young adult, −LPS: 0.06; Young adult, +LPS: 0.64; Old, −LPS: 0.39; Old, +LPS: 1.03).

Microglia activation:

Distance to endothelial cell dependence (Juvenile, −LPS: −0.00; Juvenile, +LPS: 0.28; Young adult, −LPS: 0.02; Young adult, +LPS: 0.47; Old, −LPS: 0.36; Old, +LPS: 0.48).

Distance to VLMC dependence (Juvenile, −LPS: 0.05; Juvenile, +LPS: 0.34; Young adult, −LPS: 0.04; Young adult, +LPS: 0.53; Old, −LPS: 0.37; Old, +LPS: 0.49).

Distance to oligodendrocyte dependence (Juvenile, −LPS: −0.02; Juvenile, +LPS: 0.26; Young adult, −LPS: 0.00; Young adult, +LPS: 0.48; Old, −LPS: 0.38; Old, +LPS: 0.50).

#### Cell-cell proximity analysis

To compute the proximity frequency between two sets of cells, i.e. cell type *A* and cell type *B*, first the true cell-cell proximity frequency μ_true_ was computed as follows: for each cell in cell type *A*, the average number of cells of cell type *B* were counted within a radius of 30 μm. To compute the null distribution of proximity (the probability that two cell types would be within 30 μm just by chance, given their local density), the locations of each cell of cell type *B* were then randomly jittered independently in both spatial dimensions (x and y) using a uniform distribution over the interval (−100 μm, 100 μm), and the number of cells in cell type *B* within 30 μm of each cell in cell type *A* was re-computed. This was repeated 1,000 times, to form a background distribution of the frequency of cell-cell proximity that would be expected to occur due to chance. The enrichment of cell-cell proximity between cell type *A* and cell type *B* was then computed as the log of the ratio between the true proximity frequency μ_true_ and the average background frequency μ_background_: log_2_(μ_true_/μ_background_). A p-value for this fold change was found by computing the *Z* score distribution across the 1,000 randomizations:

$$Z=(\mu_{\text{true}}-\mu_{\text{background}})/\sigma_{\text{background}}$$

P=2(1−CDF(|Z|))

The enrichment and p-value were computed for each pair of major cell types, and the resulting p-values were FDR adjusted across all cell type pairs.

#### Analysis of MERFISH data obtained from LPS-treated mice

To transfer cell type and cluster labels obtained from cells in the untreated mice to cells from mice treated with LPS, we took a supervised classification approach. First, the +LPS data were pre-processed as described earlier to remove doublets and obtain normalize, log-transformed, and Z-scored expression values. The −LPS and +LPS MERFISH data were then co-embedded in a joint principal component space using Harmony to compute the first 25 principal components. A multilayer perceptron classifier from sci-kit-learn was trained on the cell type and cluster annotations from the −LPS cells, using the first joint principal components as input. The classifier was then applied to the +LPS cells, to yield a final set of predicted cluster annotations that were used for subsequent analysis.

To identify genes differentially expression between the +LPS and −LPS conditions, for each gene in the MERFISH library, a model was fit using ordinary least squares that compared the two conditions for ~1-month (4-week) old mice. For each gene i, the average expression was modeled for cells in a given cell type in the +LPS and −LPS conditions using the following ordinary least squares model, fit to the normalize, log-transformed, *Z*-scored MERFISH expression data:

$$y_{i}\sim C(LPS)+\text{intercept}+\varepsilon$$

where C(*LPS*) is a categorical variable with the −LPS value set to be the reference level (i.e. C(−LPS) = 0) and the C(+LPS) value determined from the model. This was then compared with a null model lacking the C(*LPS*) categorical variable, i.e. *y*_*i*_ ~ intercept + *ε*, to determine the gene’s FDR-adjusted p-value. Likewise, we performed similar analysis to determine genes differentially expression between the 4-week-old and 90-week-old mice without LPS treatment from the MERFISH data. To identify whether a gene was upregulated in response to LPS, age, or both LPS and age, the C(+LPS) or C(90-week) values for each gene for each cell type were computed. These values were then Z-scored across genes, and genes with a *Z* score >2 and FDR-adjusted p-value <10^−4^ in a particular condition was determined as being significant for that condition.

## Supplementary Material

Supplementary Table 1

Supplementary Table 2

Supplementary Table 3

1

## Figures and Tables

**Table:** KEY RESOURCES TABLE

REAGENT or RESOURCE	SOURCE	IDENTIFIER
Experimental Models: Animals		
Mouse: C57BL/6J	Jackson Labs	Cat# 664; RRID:IMSR_JAX:000664
Chemicals, peptides, and recombinant proteins		
Formamide	Ambion	Cat# AM9342
20xSSC	Ambion	Cat# AM9763
Triton X-	Sigma	Cat# T8787
Glucose oxidase	Sigma	Cat# G2133
Phusion^®^ Hot Start Flex 2X Master Mix	New England Biolabs	Cat# M0536
Maxima H Minus Reverse Transcriptase	ThermoFisher	Cat# EP0752
dNTP mix	ThermoFisher	Cat# R1121
32% Paraformaldehyde	Electron Microscopy Sciences	Cat# 15714S
RNase inhibitor, Murine	New England Biolabs	Cat# M0314
1M Tris, pH 8	ThermoFisher	Cat# 15568025
Catalase	Sigma	Cat# C3155
6-hydroxy-2,5,7,8-tetramethylchroman-2-carboxylic acid (Trolox)	Sigma	Cat# 238813
Tris(2-carboxyethyl)phosphine (TCEP) HCl	GoldBio	Cat# TCEP1
Hoescht 33,342, Trihydrochloride, Trihydrate	ThermoFisher	Cat# H3570
Lipopolysaccharides from *E. coli* O 111:B4	Sigma	Cat# L4391
Yeast tRNA	ThermoFisher	Cat# AM7119
Dextran sulfate	Sigma	Cat# S4030
Ethanol	Decon Labs	Cat# V1016
SDS	ThermoFisher	Cat# 15553027
Proteinase K	New England Biolabs	Cat# P8107S
Ethylene carbonate	Sigma	Cat# 676802–1L
Glucose	Sigma	Cat# G7021
Oligonucleotides		
Readout probes	Integrated DNA Technologies	See Table S3
Encoding oligonucleotide probe library	Twist Biosciences	See Table S2
Anchor probe:/5Acryd/TTGAGTGGATGGAGTGTAATT+TT+TT + TT + TT + TT + TT + TT + TT + TT + T	Integrated DNA Technologies	N/A
Software and algorithms		
Custom analysis software	This paper	https://github.com/ZhuangLab/SpatialBrainAgingCell22
MERlin	Xia et al.^62^	https://github.com/ZhuangLab/MERlin
Scrublet	Wolock et al.^63^	https://github.com/swolock/scrublet
Anndata	Virshup et al.^64^	https://github.com/scverse/anndata
BBKNN	Polahski et al.^65^	https://github.com/Teichlab/bbknn
Harmony	Korsunsky et al.^35^	https://github.com/slowkow/harmonypy
Scanpy	Wolf et al.^66^	https://github.com/scverse/scanpy
Allcools	Liu et al.^38^	https://github.com/lhqing/ALLCools
Cellpose	Stringer et al.^67^	https://github.com/MouseLand/cellpose
Deposited data		
snRNA-seq data	This paper	GEO: GSE207848
snRNA-seq data and MERFISH data	This paper	https://cellxgene.cziscience.com/collections/31937775-0602-4e52-a799-b6acdd2bac2e

## Highlights

- Molecular and cellular architecture of aging frontal cortex and striatum is mapped
- Aging induces spatially dependent cell-state changes in non-neuronal cells
- White matter is a hotspot for aging-related glial and immune cell activation
- Aging- and LPS-induced brain inflammation exhibit similarities and differences
