# Supplementary material for: Molecular and spatial signatures of mouse brain aging at single-cell resolution
Source: Cell. Author manuscript; Available in PMC 2023 Mar 18. (PMC10024607; doi:10.1016/j.cell.2022.12.010)
Supplement: 1 [file NIHMS1881768-supplement-1.pdf]

Supplemental figures

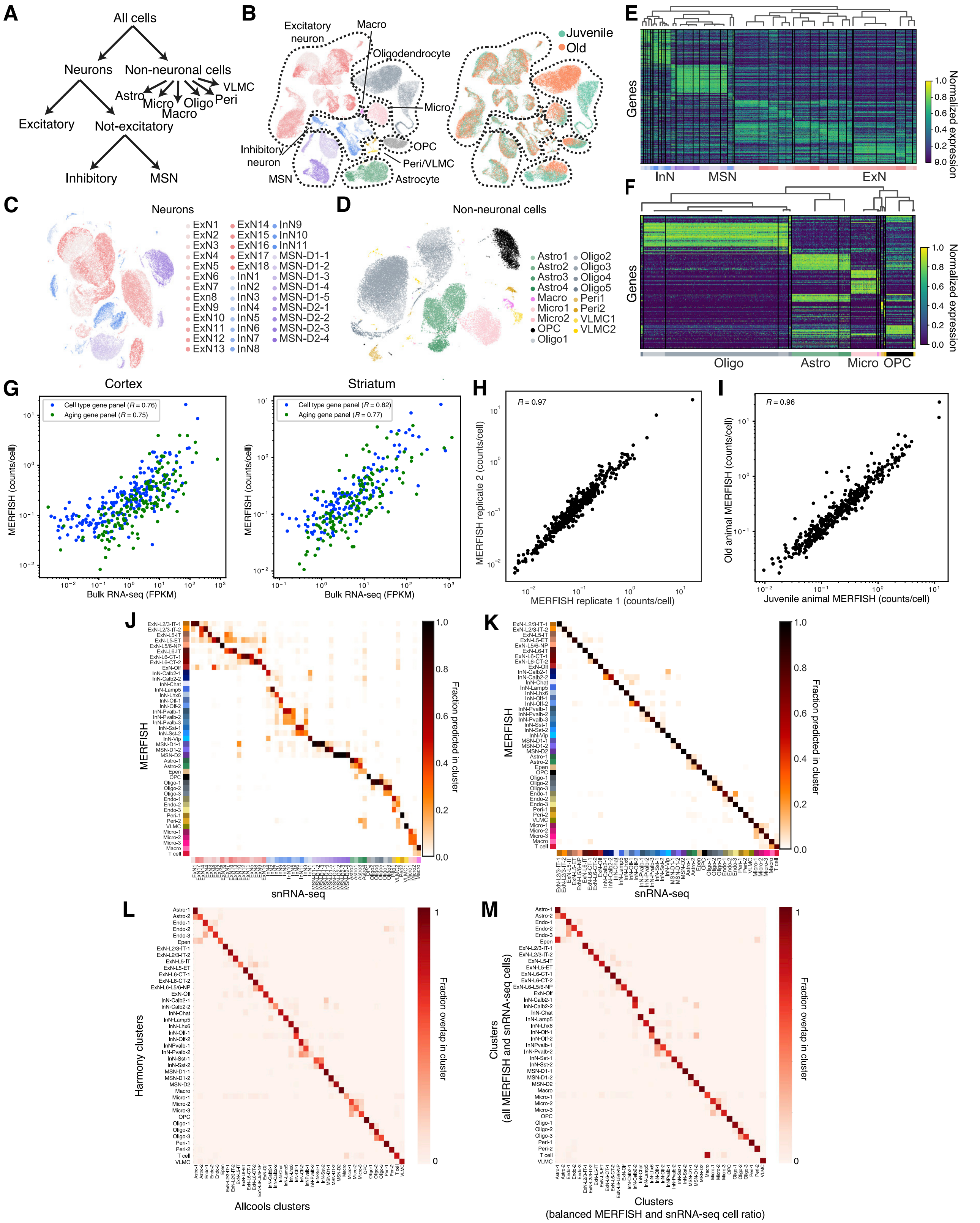

(legend on next page)

Figure S1. Clustering of cell types measured by snRNA-seq and correlation of MERFISH measurement with RNA-seq measurements, related to Figure 1

(A) Flow chart for multi-step cell clustering analysis. MSN: medium spiny neurons, Astro: astrocytes, Micro: microglia, Macro: macrophages, Oligo: oligodendrocytes, Peri: pericytes, and VLMC: vascular leptomeningeal cells.

(B) UMAP of cells colored by cell types (left) or ages (right).

(C and D) UMAP of neuronal clusters (C) and non-neuronal clusters (D).

(E and F) Heatmap of top marker genes across different neuronal clusters (E) and non-neuronal clusters (F).

(G) Correlation of average expression values of individual genes included in the cell-type marker gene panel and aging-related gene panel measured by MERFISH with FPKM values of the same genes measured by bulk RNA-seq for motor cortex[36] (left) and striatum[37] (right).

(H) Correlation of average expression values of individual genes between two biological replicates measured by MERFISH.

(I) Correlation of average expression values of individual genes between juvenile and old mice measured by MERFISH.

Pearson correlation coefficients *R* are given in (G–I).

(J) Correspondence between MERFISH clusters, determined from an integrated snRNA-seq and MERFISH clustering analysis shown in Figure 1, and snRNA-seq clusters, determined from a separate clustering analysis of the snRNA-seq data alone shown in Figures S1A–S1D.

(K) Correspondence between MERFISH and snRNA-seq clusters, both determined from the integrated MERFISH and snRNA-seq clustering analysis.

In (J) and (K), a neural network classifier was used to predict a MERFISH cluster identity for each cell measured in the snRNA-seq dataset. The fraction of cells from any given snRNA-seq cluster (columns) that was predicted to have each MERFISH cluster label (rows) was plotted.

(L) Correspondence between clusters found using the Harmony integration method and Allcools integration method. For each Harmony cluster, the fractions of cells in this cluster that belong to the Allcools clusters are shown.

(M) Correspondence between clusters found using integration of full MERFISH dataset (~400,000 cells) and full snRNA-seq dataset (~80,000 cells) and clusters found using intergration with a balanced ratio between MERFISH and scRNA-seq cells (i.e. subsampling MERFISH dataset to ~80,000 cells) using Harrmony. A few noticeable off-diagonal elements correspond to rare cell types that were not sampled well when the MERFISH cell number was reduced to 80,000, in particular, T cells, ependymal cells, and the inhibitory neuronal type, InN-Chat.

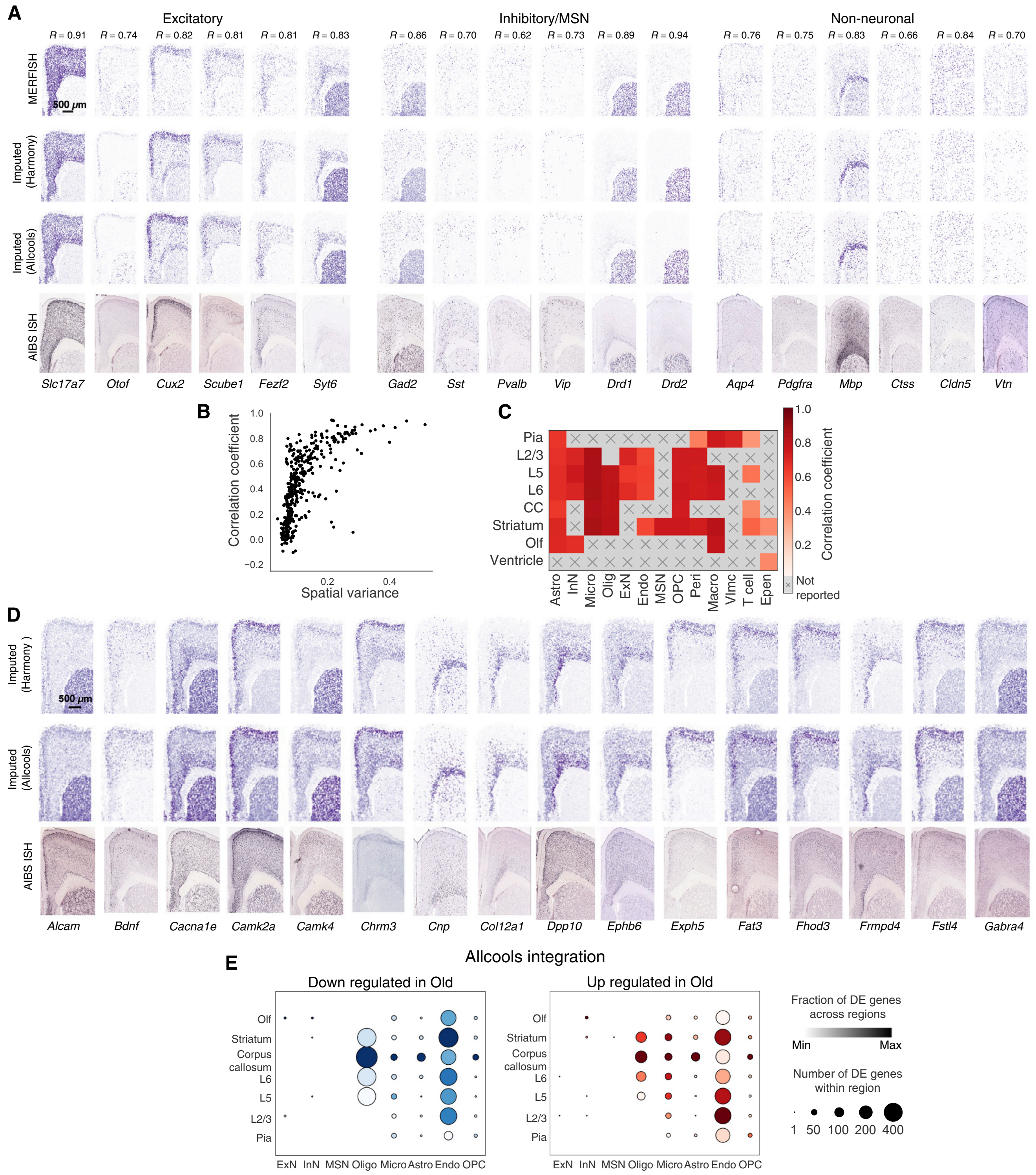

Figure S2. Comparison of imputation results with MERFISH measurement results and Allen Brain Institute *in situ* hybridization data, related to Figures 1, 2, 3, and 4

(A) Spatial plots of expression of marker genes for different excitatory, inhibitory, and medium spiny neurons, and non-neuronal cell types in measured MERFISH data, imputed gene expression data (from both Harmony and Allcools integrations), and Allen Institute for Brain Science (AIBS) *in situ* hybridization (ISH) data. The Pearson correlation coefficient, R, between the spatial map measured by MERFISH and that derived from imputation (Harmony integration) is shown for each gene. Scale bar: 500 μm.

(legend continued on next page)

(B) Scatterplot showing a positive correlation of the spatial correlation coefficient between the measured and imputed results (Harmony integration) and the spatial variance in the expression level of the gene. The spatial correlation coefficients are relatively high for essentially all genes that showed spatially heterogeneous expression and relatively low for genes that exhibited spatially uniform expression, as expected.
(C) Correlation coefficients of the gene-expression-profiles between imputed (Harmony integration) and measured results for all cell types in individual anatomic regions. For each cell type, only the anatomic regions with a cell number that is greater than 10% of the total cell number of that cell type are considered.
(D) Spatial plots of representative imputed genes that were not measured in the MERFISH gene panel and corresponding AIBS ISH data. Scale bar: 500 μm. The AIBS ISH data in (A) and (D) are taken from https://mouse.brain-map.org/ (credit: Allen Institute).
(E) As in Figure 4E, but using imputed gene expression data derived from Allcools integration.

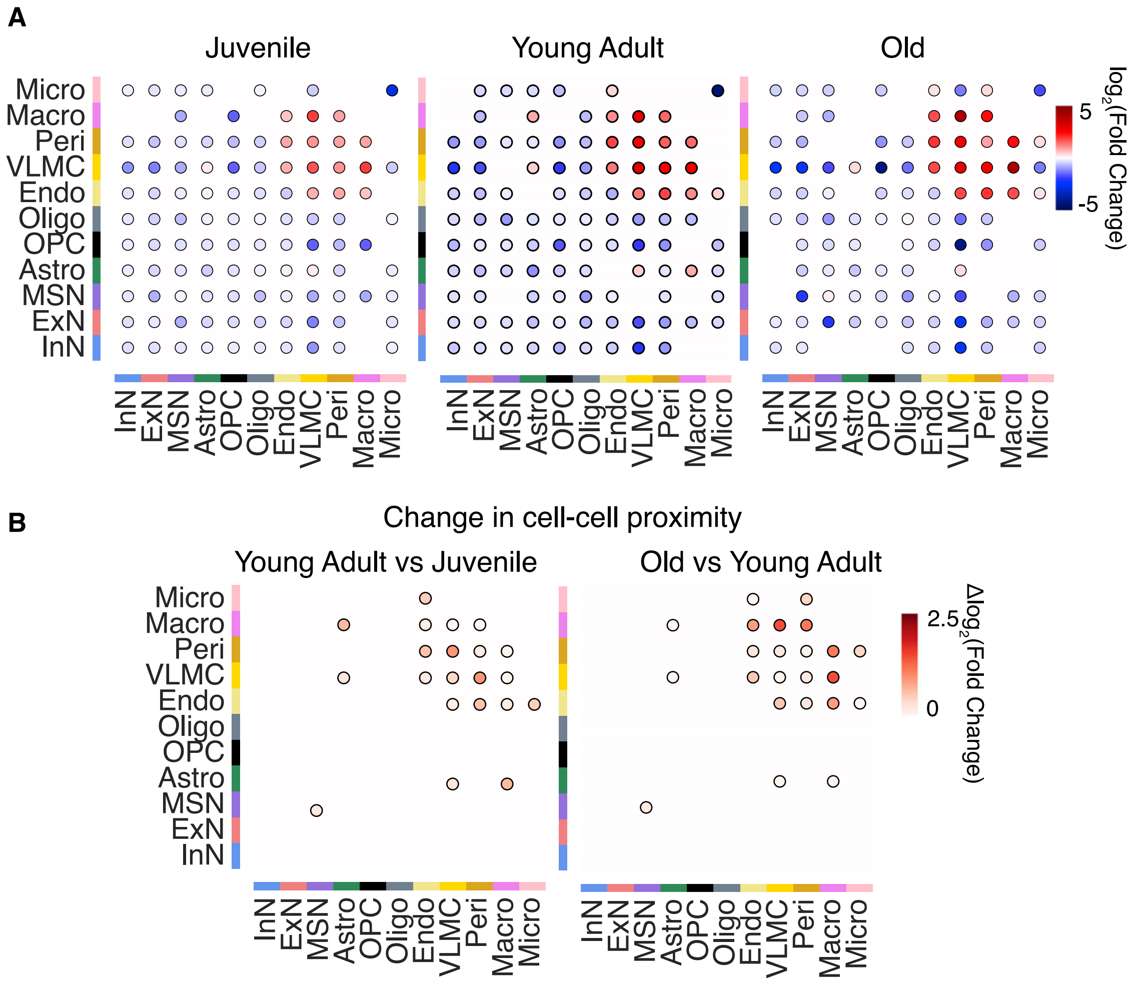

Figure S3. Cell-cell proximity between different cell types across three different ages, related to Figure 3

(A) Enrichment of cell-cell proximity between different cell-types across three different ages. Enrichment in proximity between cell-type pairs was computed as describe in STAR Methods. Plots show only cell-type pairs with statistically significant enrichment (FDR-adjusted *p* value <0.05).

(B) Difference in enrichment of cell-cell proximity between young adult and juvenile animals (left) and between old and young adult animals (right).

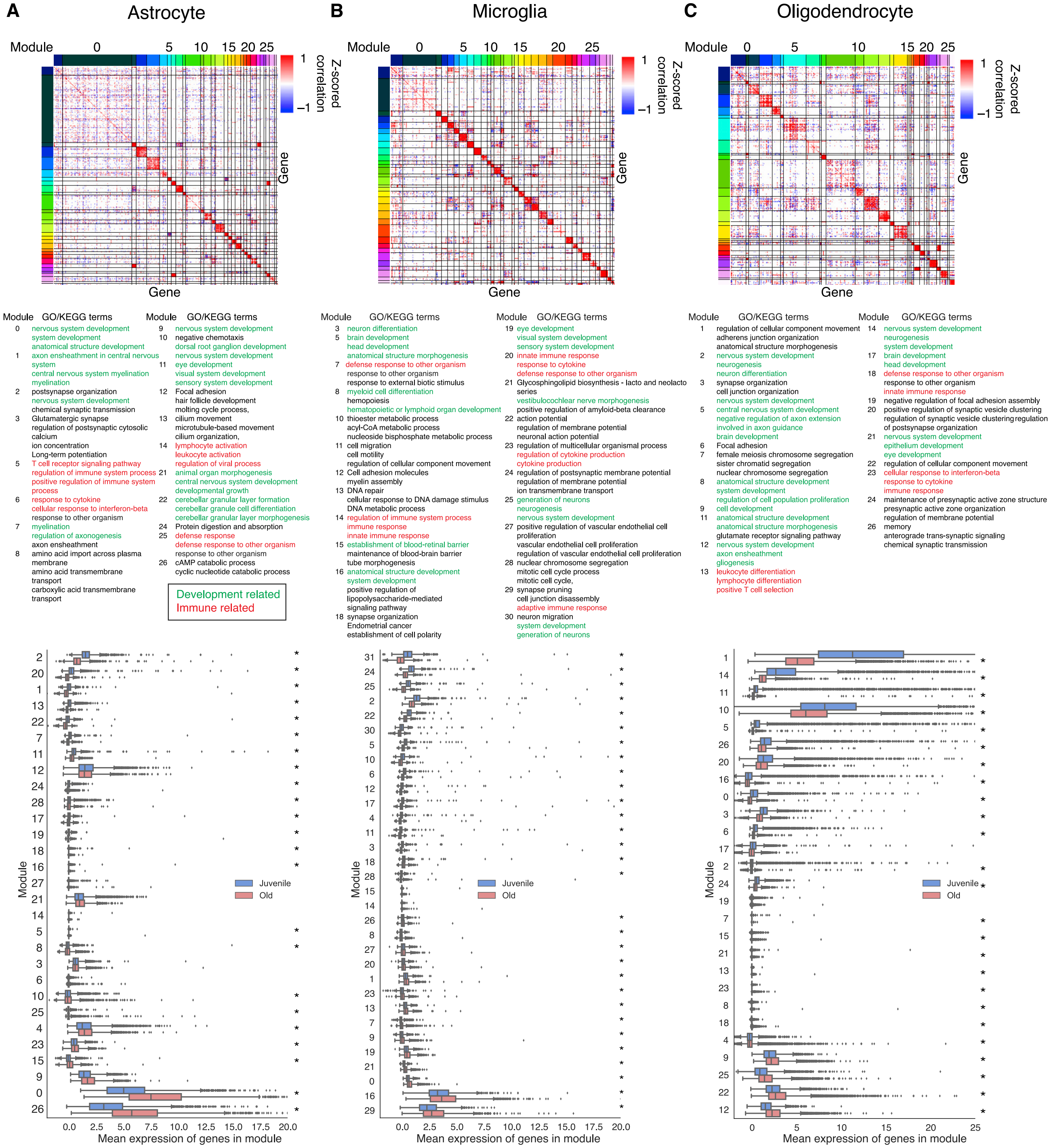

Figure S4. Gene modules analysis for astrocytes, microglia, and oligodendrocytes, related to Figure 4

(A, B and C) Top: Gene-gene correlation matrix for variable genes in astrocytes (A), microglia (B), and oligodendrocytes (C). Z-scored Pearson correlation coefficient for the expression levels of pairs of genes are shown and ordered by hierarchical clustering to show groups of genes with correlated expression, which we termed gene modules. Middle: Top Gene Ontology (GO) or Kyoto Encyclopedia of Genes and Genomes (KEGG) terms enriched in modules (up to three terms from the top five terms are shown per module). Only modules with any enriched terms are shown. Development-related terms are colored green and immune-related terms are colored red. Bottom: Mean expression level of the genes in each module in juvenile and old mice, sorted by average difference between juvenile and old. Boxplots show the distribution across individual cells, with the box showing median and interquartile range, whiskers showing minimum and maximum, and outliers shown as fliers. * indicates FDR-adjusted p value <0.05 (independent t-test).

CellPress
OPEN ACCESS

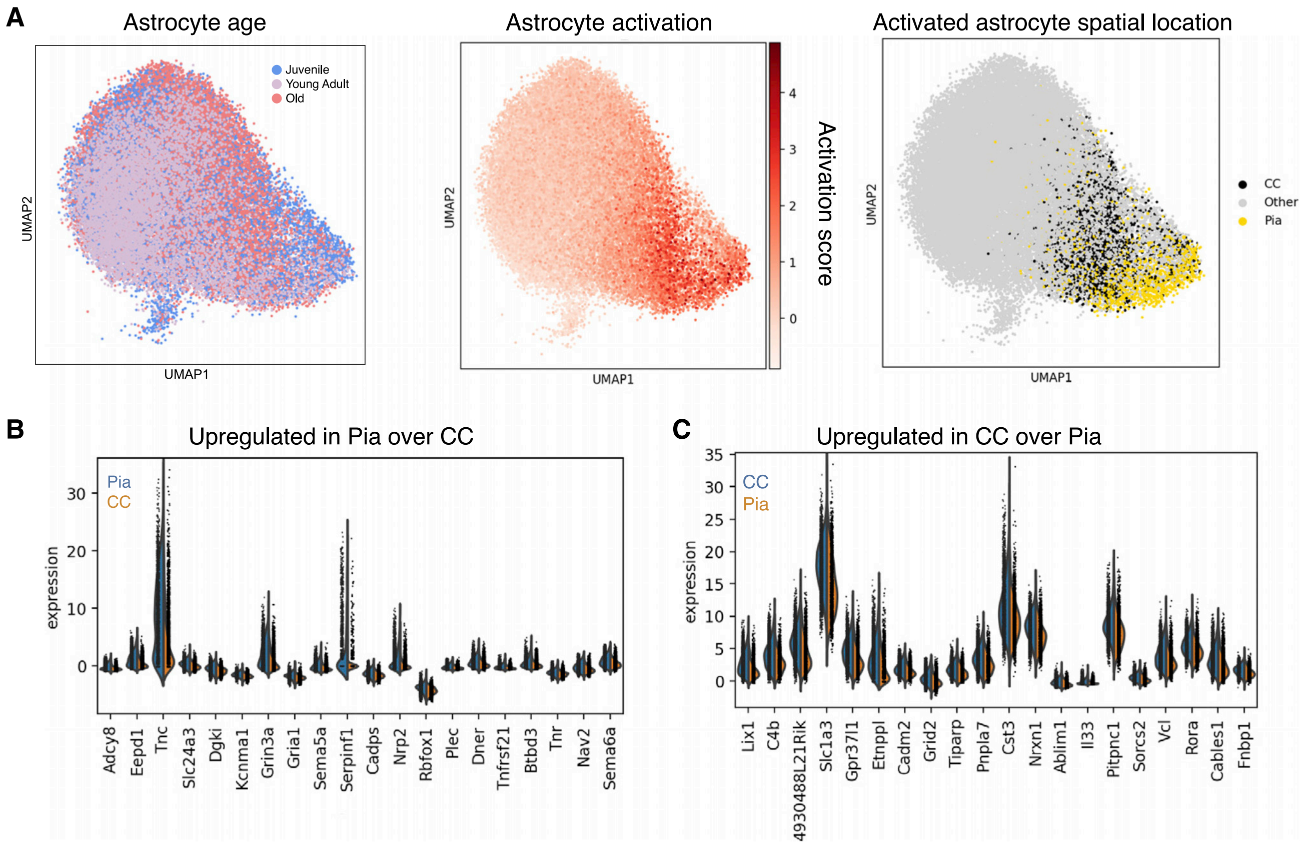

Figure S5. Molecular heterogeneity of activated astrocytes in pia and corpus callosum, related to Figure 5

(A) UMAP of astrocytes colored by the age of cells (left), by the activation score of cells (middle), and by the spatial location of activated cells (Right; black: activated astrocytes in corpus callosum [CC]; Yellow: activated astrocytes in pia: Gray: all other astrocytes).

(B and C) Violin plots of relative expression levels of differentially expressed genes between activated astrocytes in pia vs corpus callosum, showing genes that are upregulated in pia relative to corpus callosum (B) and upregulated in corpus callosum vs pia (C). Activated cells were defined as having activation score greater than 1 SD above the mean.

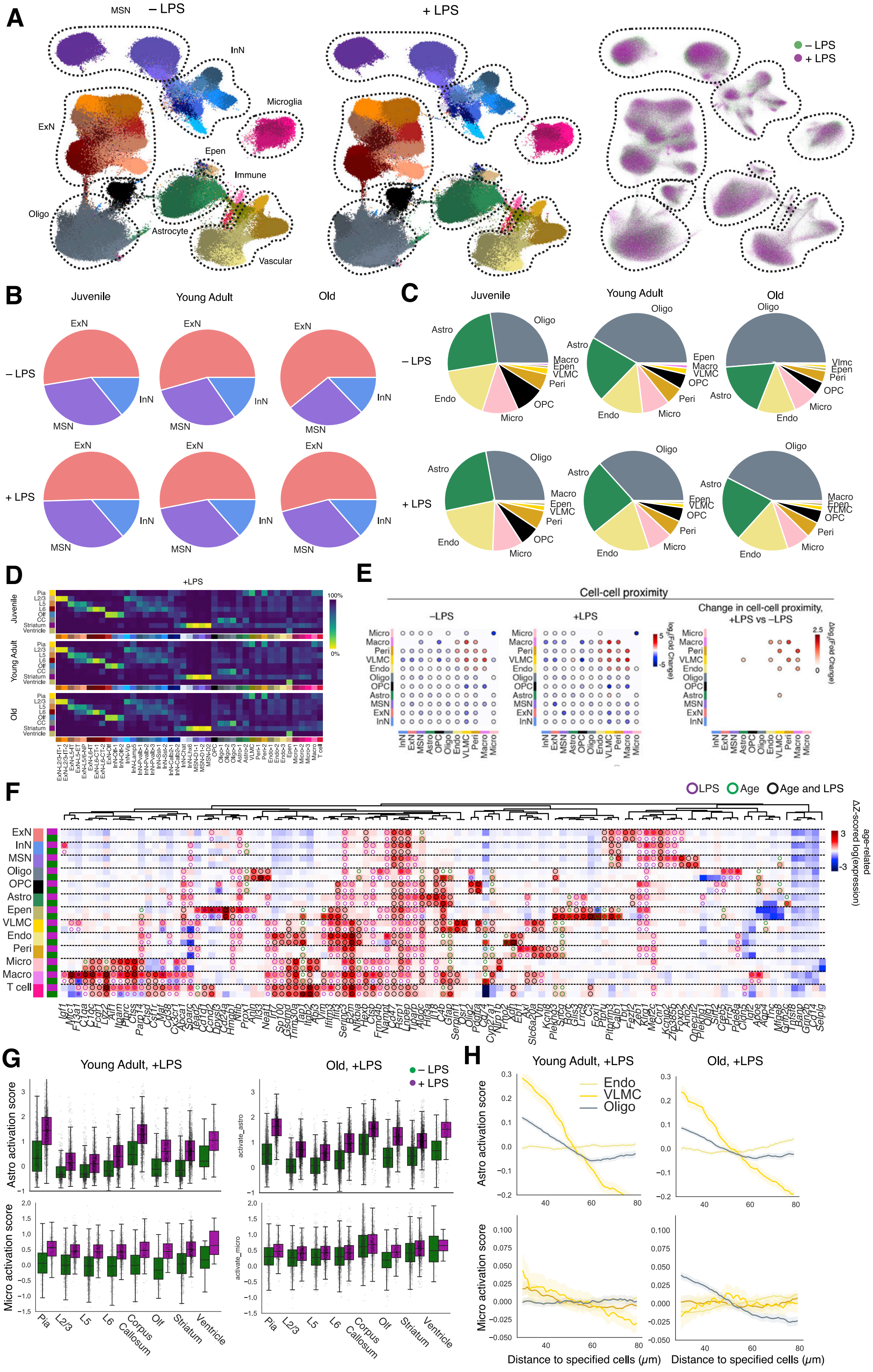

(legend on next page)

Figure S6. Comparison of cell-type compositions and spatial organization between –LPS and +LPS treatment conditions, and cell-type-specific changes in response to LPS treatment, related to Figure 6

(A) (Left) Integrated UMAP of cells measured in –LPS and +LPS conditions. Cells are colored by their cluster identities. (Right) Overlay of cells colored by –LPS or +LPS condition.

(B and C) Major neuronal (B) and non-neuronal (C) cell-type composition across the three different ages, for –LPS and +LPS conditions.

(D) Fraction of cells in different anatomical regions in +LPS condition across three different ages, as in Figure 3B.

(E) (Left) Enrichment of cell-cell proximity between different cell types for –LPS and +LPS conditions in juvenile animals, as in Figure S3. (Right) Difference in enrichment of cell-cell proximity between –LPS and +LPS conditions in juvenile animals.

(F) Changes in expression of individual genes for each cell type, where alternating rows show the LPS-related change in Z-scored log(gene expression) (comparing +LPS vs. –LPS, juvenile mice) and age-related changes (comparing juvenile vs old mice, –LPS). Green, magenta, and black circles respectively mark genes substantially upregulated with age, LPS, or both age and LPS, as in Figure 6C.

(G) Per-cell activation scores for microglia and astrocytes in different anatomical regions in young adult (left) and old (right) animals with LPS treatment. Boxplots are as defined in Figure 5D.

(H) Activation scores of astrocytes and microglia as a function of distance from neighboring oligodendrocytes, VMLCs, and endothelial cells in young adult (left) and old (right) animals after LPS treatment, as in Figure 5E. Data are presented as mean (solid line) ± SEM (shade).

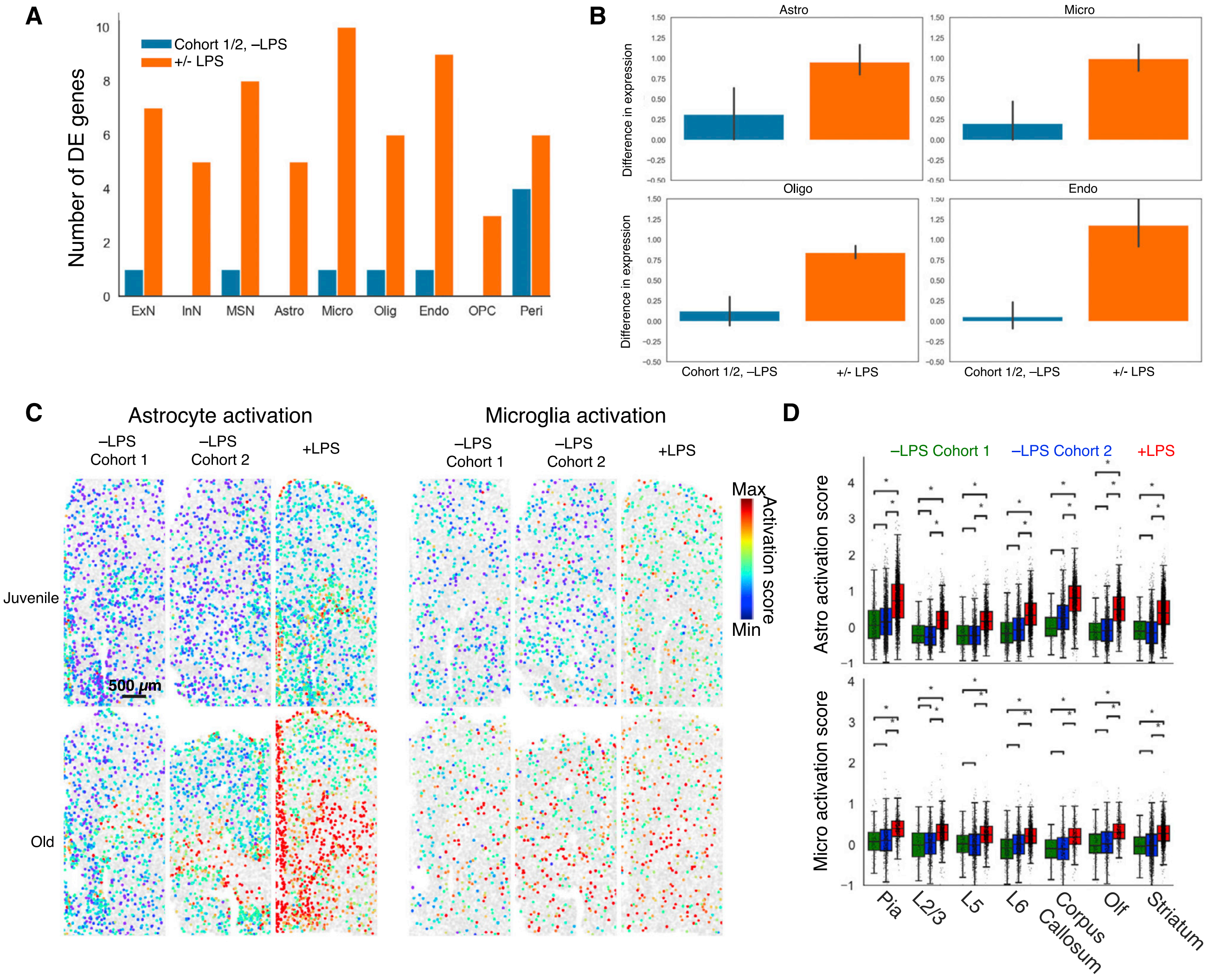

Figure S7. Comparison of LPS treatment effect with animal cohort-to-cohort variation, related to Figure 6

(A) Blue: Number of differentially expressed (DE) genes (change in Z-scored log(gene expression >2, FDR-adjusted P-value <0.01) between two juvenile animal cohorts in the –LPS condition within each major cell type. Orange: Number of DE genes between –LPS and +LPS conditions within each major cell type in juvenile mice.

(B) Average expression-level change of the DE genes between +LPS and –LPS conditions (orange) or between two animal cohorts in the –LPS condition (blue) for astrocytes, microglia, oligodendrocytes, and endothelial cells. Data are presented as mean ±95% confidence interval.

(C) Spatial plot of astrocyte and microglia activation in juvenile and old mice in the –LPS condition and +LPS condition. Results from two animal cohorts are show for the –LPS condition. Scale bar: 500 μm.

(D) Activation scores of astrocytes and microglia in different anatomical regions in juvenile mice in the –LPS condition (blue: animal cohort 1; green: animal cohort 2) and +LPS condition (red). * marks statistically significant changes (Bonferroni-adjust p value $<10^{-6}$, t-test). Boxplots are as defined in Figure 5D.
